# KLHL29-mediated DDX3X degradation promotes chemosensitivity by abrogating cell cycle checkpoint in triple-negative breast cancer

**DOI:** 10.1038/s41388-023-02858-5

**Published:** 2023-10-16

**Authors:** Litong Yao, Qian Hao, Mozhi Wang, Yuhai Chen, Hongyi Cao, Qiang Zhang, Keda Yu, Yizhou Jiang, Zhiming Shao, Xiang Zhou, Yingying Xu

**Affiliations:** 1 Department of Breast Surgery, the First Hospital of China Medical University, Shenyang, Liaoning China; 2 Fudan University Shanghai Cancer Center, Fudan University, Shanghai, China; 3 Department of Pathology, the First Hospital of China Medical University and College of Basic Medical Sciences, Shenyang, Liaoning China; 4 Department of Breast Surgery, Cancer Hospital of China Medical University, Liaoning Cancer Hospital & Institute, Shenyang, Liaoning China; 5 Shanghai Key Laboratory of Medical Epigenetics, International Co-laboratory of Medical Epigenetics and Metabolism (Ministry of Science and Technology), Institutes of Biomedical Sciences, Fudan University, Shanghai, China

**Keywords:** Breast cancer, Ubiquitylation, Predictive markers

## Abstract

Triple-negative breast cancer (TNBC) is a heterogeneous breast cancer subtype and accounts for approximately 15–20% of breast cancer cases. In this study, we identified KLHL29, which is an understudied member of the Kelch-like gene family, as a crucial tumor suppressor that regulates chemosensitivity in TNBC. KLHL29 expression was significantly downregulated in breast cancer tissues compared with adjacent normal tissues, and low levels of KLHL29 were associated with unfavorable prognoses. Ectopic KLHL29 suppressed, while depleting KLHL29 promoted, the growth, proliferation, migration, and invasion of TNBC. Mechanistically, KLHL29 recruited the CUL3 E3-ligase to the RNA-binding protein DDX3X, leading to the proteasomal degradation of the latter. This downregulation of DDX3X resulted in the destabilization of CCND1 mRNA and the consequent cell cycle arrest at G0/G1 phase. Remarkably, the DDX3X inhibitor RK33 combined with platinum-based chemotherapy can synergistically suppress TNBC that usually expresses low levels of KLHL29 and high levels of DDX3X using cancer cell-derived xenograft and patient-derived organoids models. Altogether, we uncovered the potential role for the KLHL29-DDX3X signaling cascade in the regulation of TNBC progression, thus providing a promising combination strategy for overcoming TNBC chemoresistance.

## Introduction

Breast cancer is the most common malignancy and the leading cause of cancer-related mortality among females worldwide [1]. Triple-negative breast cancer (TNBC) accounts for approximately 15–20% of breast cancer cases and is defined by the negative expression of estrogen receptor, progestogen receptor (PR) and human epidermal growth factor receptor 2 (HER2) [2–4]. Representing a subgroup of biologically and clinically heterogeneous breast cancer, TNBC is characterized by larger tumor size, higher tumor grade, higher proliferative capacity, and, more likely, lymph node involvement [5, 6]. Patients with TNBC do not benefit from established endocrine or HER2-targeted therapy due to the lack of related receptor markers [7]. More recently, several novel agents including immunotherapy, targeted therapy, and antibody-drug conjugates are constantly being introduced for the treatment of TNBC and have transformed the treatment landscape of TNBC [8]. Conventional cytotoxic chemotherapy based on anthracyclines and taxanes continues to be the backbone of standard of care. However, a considerable number of patients exhibit limited responsiveness or eventually develop drug resistance [9, 10]. Because of the highly aggressive nature of TNBC and the absence of effective therapeutics and predictive biomarkers, elucidating the mechanisms underlying TNBC progression and chemoresistance remains an active area of investigation.

Our previous work illustrated the genomic, transcriptomic and proteomic landscape of TNBCs in Chinese patients treated at Fudan University Shanghai Cancer Center (FUSCC) to provide a valuable molecular infrastructure for further clinical investigation and improve the understanding of molecular mechanisms of TNBC [11, 12]. Accordingly, we identified multiple TNBC-driving genes that may serve as potential therapeutic targets. For instance, decaprenyl diphosphate synthase subunit 1 (PDSS1) promotes TNBC metastasis by regulating the CAMK2A-STAT3 signaling pathway [13]. Alpha-endosulfine (ENSA) enhances cholesterol biosynthesis by increasing the phosphorylation of STAT3 and thus the expression of SREBP2, a pivotal transcription factor driving the mevalonate pathway [14]. A tumor-specific splicing variant of macrophage receptor with collagenous structure (MARCO) promotes metabolic dysregulation resulting in a hypoxic tumor microenvironment by regulating the HIF-1α signaling [15]. Characterization of these oncogenic proteins offers insights into the biological underpinnings in TNBC. In this study, we sought to identify tumor suppressors based on the multi-omics data from our TNBC cohort, resulting in the identification of KLHL29, which is an understudied member of the Kelch-like gene family.

The Kelch-like (KLHL) gene family encodes a group of evolutionarily conserved proteins that usually harbor a BTB/POZ domain, a BACK domain, and five to six Kelch motifs [16]. Members of the KLHL family are found to participate in a variety of cellular processes, such as mitotic progression, DNA damage repair, autophagy, and apoptosis, by connecting the E3-ubiquitin ligase Cullin 3 (CUL3) and various proteins substrates [17, 18]. Furthermore, several KLHL proteins have been shown to play important roles in tumorigenesis and cancer progression [19–22]. Here, we identified one of the KLHL family members, KLHL29, as a novel tumor suppressor gene in TNBC. The expression of KLHL29 was significantly downregulated in cancerous tissues compared with normal tissues and low levels of KLHL29 were associated with unfavorable prognoses in TNBC. Ectopic KLHL29 suppressed TNBC cell proliferation, migration, and invasion, whereas depleting KLHL29 promoted TNBC progression. Mechanistically, KLHL29 acted as a specific adapter mediating the ubiquitination and proteasomal degradation of the RNA-binding protein DDX3X via CUL3, consequently leading to cell cycle arrest at G0/G1 phase. Remarkably, we showed that platinum-based chemotherapy combined with the DDX3X inhibitor, RK33, effectively suppressed TNBC growth in vitro and in vivo. Collectively, our study uncovers a tumor suppressive role for KLHL29 in TNBC and provides a promising combination strategy for overcoming TNBC chemoresistance.

## Results

### KLHL29 downregulation is associated with unfavorable prognosis in triple-negative breast cancer

**Fig. 1:**
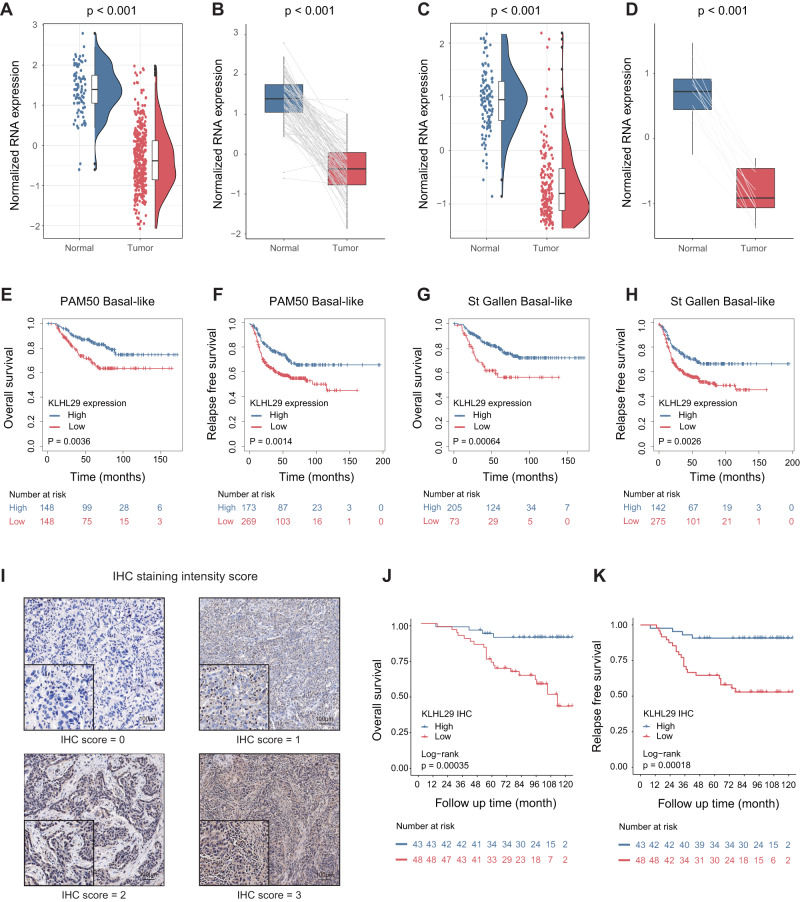
KLHL29 is downregulated in TNBC, and its downregulation is associated with unfavorable prognosis. **A** mRNA expression levels of KLHL29 in 360 TNBC tissues and 88 adjacent normal tissues in FUSCC-TNBC RNA-seq dataset. **B** mRNA expression levels of KLHL29 in 88 TNBC tissues and paired adjacent normal breast tissues in FUSCC-TNBC RNA-seq dataset. **C** mRNA expression levels of KLHL29 in 169 TNBC tissues and 113 adjacent normal tissues in TCGA TNBC RNA-seq dataset. **D** mRNA expression levels of KLHL29 in 13 TNBC tissues and paired adjacent normal breast tissues in TCGA TNBC RNA-seq dataset. Data are represented as mean ± SD. Wilcoxon rank-sum test. **E**–**H** Kaplan–Meier plots of KLHL29 in basal-like breast cancer according to PAM50 and St Gallen subtypes (https://kmplot.com/analysis/). Log-rank test. **I** Immunohistochemical staining analysis of KLHL29 expression in TNBC specimens. Representative images are shown. **J**, **K** Low expression level of KLHL29 is associated with poor overall survival (**J**) and relapse-free survival (**K**) Log-rank test.

By analyzing the multi-omics data from the TNBC cohort at FUSCC [11], we identified an understudied KLHL family gene, KLHL29, whose mRNA levels were significantly downregulated in TNBC tissues compared with adjacent normal tissues (Fig. 1A). This correlation was also observed in the 88 paired samples (Fig. 1B). Consistently, the expression of KLHL29 was also downregulated in TNBC samples in TCGA database (Fig. 1C, D). We collected six pairs of primary TNBC specimens and matched adjacent normal tissues to detect the protein levels of KLHL29 by western blotting analysis. As presented in Supplementary Fig. S1A, KLHL29 was downregulated in TNBC tissues compared with normal tissues. We then evaluated the prognostic significance of KLHL29 expression in patients with TNBC. Kaplan–Meier survival analysis indicated that low expression of KLHL29 was associated with poor overall survival and relapse-free survival in basal-like breast cancer from the Kaplan–Meier plotter database (Fig. 1E–H). In addition, we collected surgical samples from 91 TNBC patients at the First Hospital of China Medical University and performed immunohistochemistry (IHC) staining analysis of KLHL29 expression (Fig. 1I). Kaplan–Meier analysis revealed that TNBC tumors with lower levels of KLHL29 were linked to worse overall survival (Fig. 1J) and relapse-free survival (Fig. 1K). Multivariate cox regression analysis again confirmed that low expression of KLHL29 is an independent risk factor for poor prognosis in patients with TNBC (Supplementary Tables S1 and S2). Together, these results indicate that KLHL29 is downregulated in TNBC and its downregulation is associated with unfavorable prognosis.

### KLHL29 suppresses cell proliferation, migration, and invasion in triple-negative breast cancer

**Fig. 2:**
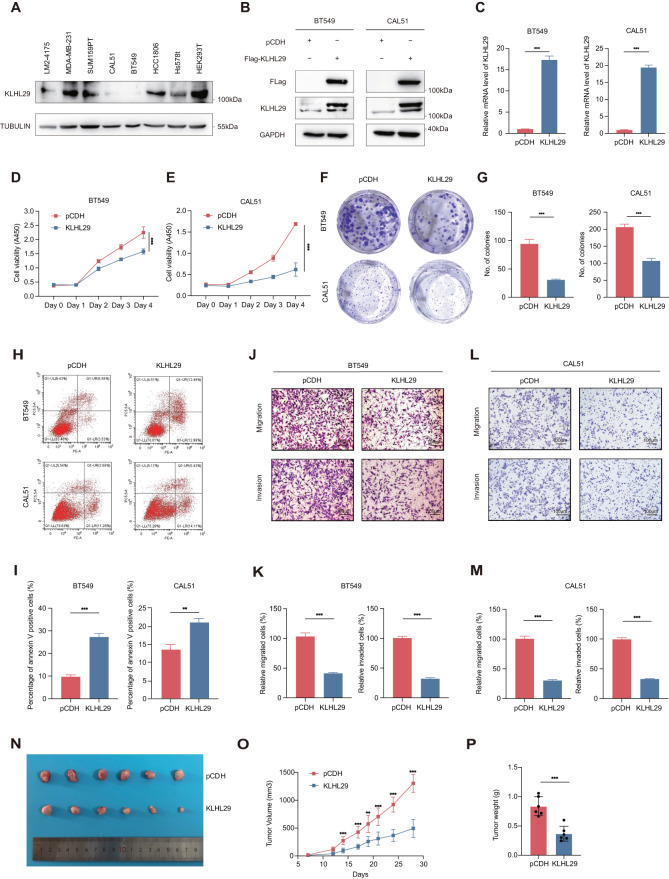
KLHL29 inhibits TNBC proliferation, migration, and invasion. **A** Western blotting analysis of endogenous protein levels of KLHL29 in TNBC cell lines and HEK293T. **B**, **C** KLHL29 expression was confirmed in BT549 and CAL51 cells stably expressing the empty vector pCDH-Flag and pCDH-Flag-KLHL29 by western blotting (**B**) and RT-qPCR (**C**) analyses. **D**, **E** Ectopic expression of KLHL29 suppresses cell proliferation of BT549 (**D**) and CAL51 (**E**) cells by cell viability assays using the Cell Counting Kit-8. **F**, **G** Ectopic expression of KLHL29 impedes colony-forming ability of BT549 and CAL51 cells by colony formation assays. Representative images of the colonies (**F**) and corresponding quantitative results (**G**) are shown. **H**, **I** Ectopic expression of KLHL29 augments cell apoptosis of BT549 and CAL51 cells by flow cytometry analysis. Representative images of cell apoptosis (**H**), and percentage of annexin V positive cells (**I**) are shown. **J**–**M** Ectopic expression of KLHL29 impairs cell migration and invasion of BT549 and CAL51 cells by cell migration and invasion assays using transwell chambers coated without and with Matrigel, respectively. Representative images of migrated and invaded cells (**J** and **L**), and corresponding quantitative results (**K** and **M**) are shown. **N**–**P** Ectopic expression of KLHL29 suppresses the growth of MDA-MB-231 cell-derived xenograft tumors. MDA-MB-231 cells stably expressing pCDH-Flag and pCDH-Flag-KLHL29 were injected orthotopically into mammary fat pad of NOD/SCID female nude mice (*n* = 6 mice per group). Representative tumor images (**N**), tumor growth rate (**O**), and tumor weight (**P**) are shown. **p < 0.01; ***p < 0.001 by two-tailed Student’s *t* test or two-way ANOVA test.

To determine the biological functions of KLHL29 in TNBC, we first analyzed the KLHL29 protein expression levels in several well-characterized TNBC cell lines (BT549, CAL51, SUM159PT, MDA-MB-231, LM2-4175, Hs578T, and HCC1806), and human embryonic kidney cell line (HEK293T) by western blotting analysis (Fig. 2A). Then, we generated KLHL29-stably overexpressing BT549 and CAL51 cells via lentiviral infection, as these two cell lines express very low levels of endogenous KLHL29. The overexpression of exogenous KLHL29 in the two cell lines was confirmed at both protein and mRNA levels by western blotting and qRT-PCR analysis (Fig. 2B, C). The cell viability assays showed that ectopic expression of KLHL29 suppressed TNBC cell proliferation (Fig. 2D, E). In addition, the results of the colony formation assays suggested that ectopic KLHL29 inhibited the colony-forming ability of TNBC cells (Fig. 2F, G). Flow cytometry analysis showed that overexpression of KLHL29 augmented apoptosis (Fig. 2H, I) of TNBC cells. Considering the highly invasive and metastatic potential of TNBC cells, we also assessed the role of KLHL29 in cell migration and invasion. Indeed, the transwell assay showed that ectopic KLHL29 impaired the migration and invasion of TNBC cells (Fig. 2J–M). We also generated KLHL29-stably overexpressing MDA-MB-231 cells (Supplementary Fig. S1B, S1C), as this TNBC cell line is highly aggressive. Consistently with the results from BT549 and CAL51 cells, ectopic expression of KLHL29 also suppressed the proliferation of these cells (Supplementary Fig. S1D). Thus, we performed a mouse xenograft experiment using MDA-MB-231 cells that overexpress KLHL29 or an empty vector. The overexpression of KLHL29 dramatically reduced the growth of MDA-MB-231 cell-derived xenograft tumors, as measured by the tumor size (Fig. 2N), growth rate (Fig. 2O), and weight (Fig. 2P). IHC staining showed the KLHL29-overexpressing samples had weaker Ki67 staining than the control samples (Supplementary Fig. S1E). These results demonstrate that KLHL29 functions as a tumor suppressor to inhibit TNBC growth and progression.

### Ablation of KLHL29 promotes cell proliferation, migration, and invasion in triple-negative breast cancer

**Fig. 3:**
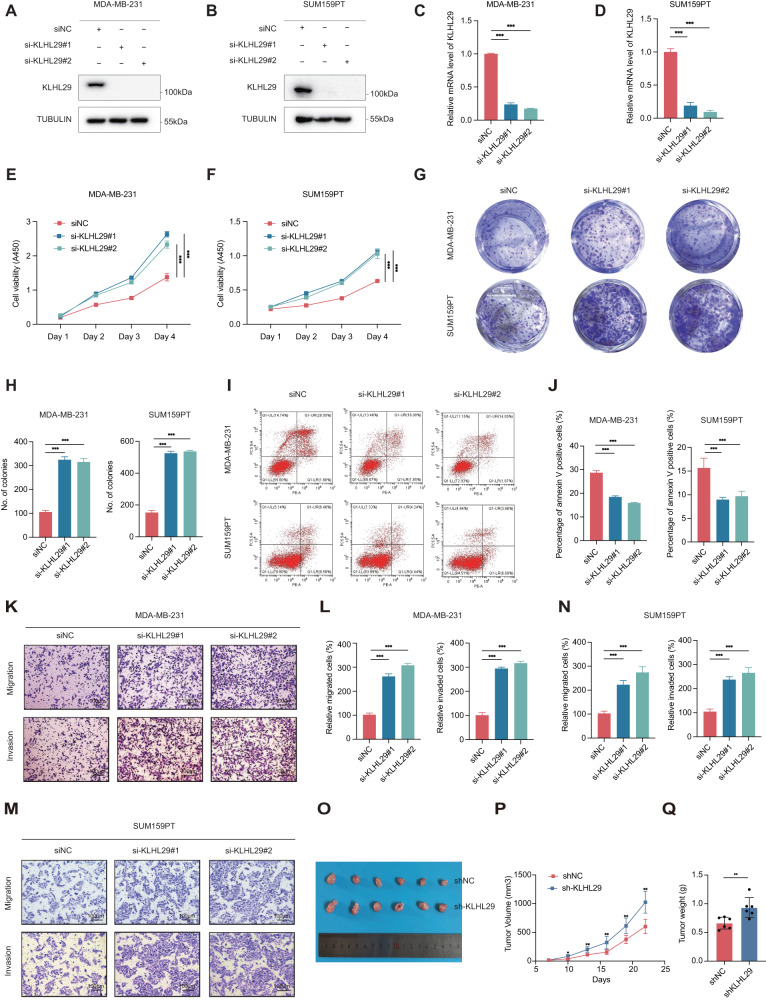
KLHL29 deficiency promotes TNBC proliferation, migration, and invasion. **A**, **B** KLHL29 expression was confirmed in MDA-MB-231 (**A**) and SUM159PT (**B**) cells transfected with two independent siRNAs targeting KLHL29 by western blotting analysis. **C**, **D** KLHL29 expression was confirmed in MDA-MB-231 (**C**) and SUM159PT (**D**) cells transfected with two independent siRNAs targeting KLHL29 by RT-qPCR analysis. **E**, **F** Ablation of KLHL29 promotes cell proliferation of MDA-MB-231 (**E**) and SUM159PT (**F**) cells by cell viability assays using the Cell Counting Kit-8. **G**, **H** Ablation of KLHL29 enhances colony-forming ability of MDA-MB-231 and SUM159PT cells by colony formation assays. Representative images of the colonies (**G**) and corresponding quantitative results (**H**) are shown. **I**, **J** Ablation of KLHL29 inhibits cell apoptosis of MDA-MB-231 and SUM159PT cells by flow cytometry analysis. Representative images of cell apoptosis (**I**), and percentage of annexin V positive cells (**J**) are shown. **K**–**N** Ablation of KLHL29 promotes cell migration and invasion of MDA-MB-231 and SUM159PT cells by cell migration and invasion assays using transwell chambers coated without and with Matrigel, respectively. Representative images of migrated and invaded cells (**K** and **M**), and corresponding quantitative results (**L** and **N**) are shown. **O**–**Q** Ablation of KLHL29 promotes the growth of MDA-MB-231 cell-derived xenograft tumors. MDA-MB-231 cells stably expressing shNC and shKLHL29 were injected orthotopically into mammary fat pad of NOD/SCID female nude mice (*n* = 6 mice per group). Representative tumor images (**O**), tumor growth rate (**P**), and tumor weight (**Q**) are shown. **p < 0.01; ***p < 0.001 by two-tailed Student’s *t* test or two-way ANOVA test.

Next, we determined the functions of endogenous KLHL29 by knocking down this gene by two independent siRNAs in MDA-MB-231 and SUM159PT cells (Fig. 3A–D). Ablation of KLHL29 dramatically enhanced cell proliferation (Fig. 3E, F) and colony formation (Fig. 3G, H), while inhibited apoptosis (Fig. 3I, J) in both MDA-MB-231 and SUM159PT cell lines. In addition, KLHL29 knockdown increased the migratory and invasive potential of these two cell lines (Fig. 3K–N). Consistently, stably knocking down KLHL29 enhanced the growth of MDA-MB-231 cells (Supplementary Fig. S1F–S1H), whereas re-expression of KLHL29 in these cells completely restored cell growth and migration (Supplementary Fig. S2A–S2D). Furthermore, the tumor suppressive role of KLHL29 was validated using a xenograft tumor model, as depleting KLHL29 significantly increased the size (Fig. 3O), growth rate (Fig. 3P), and weight (Fig. 3Q) of xenograft tumors. IHC staining showed that the KLHL29-depleting samples had stronger Ki67 staining than the control samples (Supplementary Fig. S1I). These results demonstrate that loss of KLHL29 promotes the growth and progression of TNBC.

### KLHL29 interacts with and inhibits the expression of DDX3X

**Fig. 4:**
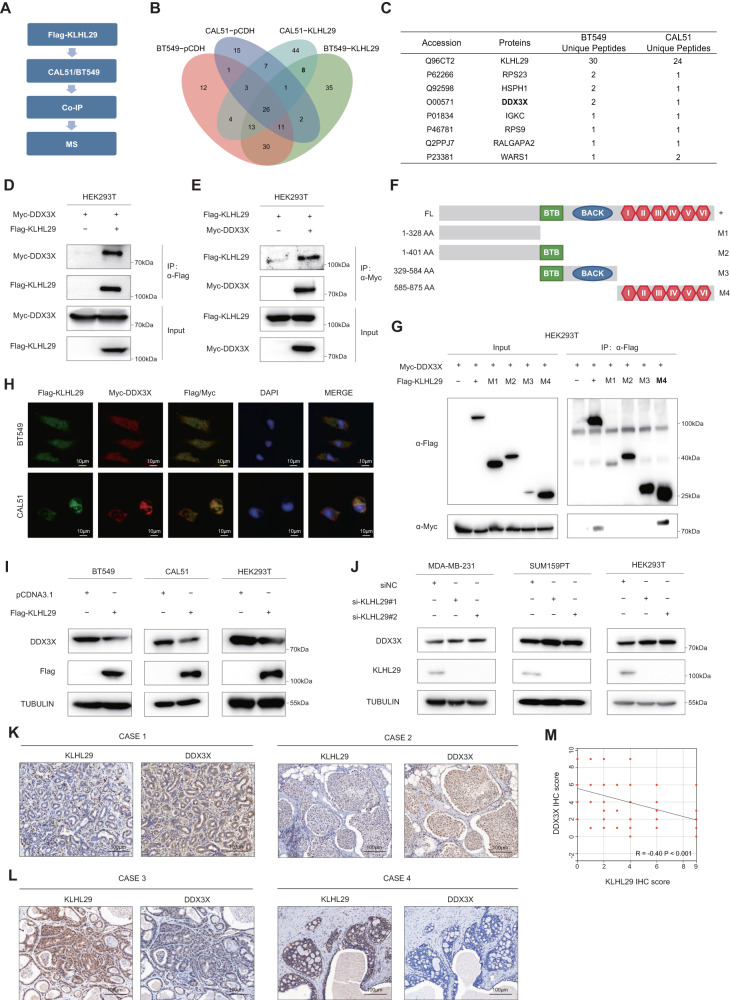
KLHL29 interacts with DDX3X and inhibits protein levels of DDX3X in TNBC. **A** The flow chart for identification of KLHL29-interacting proteins. BT549 and CAL51 cells stably expressing pCDH-Flag or pCDH-Flag-KLHL29 were subjected to IP assays, followed by MS analysis. **B** The Venn diagram analysis of KLHL29-interacting proteins. 46 proteins interact with KLHL29 in BT549 cells, and 69 proteins interact with KLHL29 in CAL51 cells. **C** The list of eight potential KLHL29-interacting proteins in both BT549 and CAL51 cells. **D**, **E** Exogenous KLHL29 interacts with exogenous DDX3X. HEK293T cells were transfected with plasmids encoding Flag-KLHL29 and Myc-DDX3X. Whole-cell lysates were immunoprecipitated with an anti-Flag or anti-Myc antibody and subjected to western blotting analysis with indicated antibodies. **F** Schematic diagram of the different KLHL29 truncation constructs used. **G** DDX3X interacts with the Kelch domain of KLHL29. HEK293T cells were transfected with the plasmids encoding the Flag-KLHL29 fragments along with the Myc-DDX3X plasmid, followed by a set of co-IP and western blotting assays. **H** Immunofluorescent images show the co-localization of Flag-KLHL29 (green) and Myc-DDX3X (red) in BT549 and CAL51 cells. The nuclear was counterstained with DAPI (blue). **I** Overexpression of KLHL29 reduces DDX3X protein levels in BT549 (left), CAL51 (middle) and HEK293T (right) cells. **J** Knockdown of KLHL29 elevates DDX3X protein levels in MDA-MB-231 (left), SUM159PT (middle) and HEK293T (right) cells. **K**, **L** Immunohistochemical staining analysis of KLHL29 and DDX3X expression in TNBC specimens. Representative images are shown. **M** Negative correlation between KLHL29 and DDX3X expression levels in TNBC specimens. Correlation coefficients were performed using the Spearman test. Two-tailed *P*-values were calculated.

To address the molecular mechanism underlying the tumor suppressive functions of KLHL29 in TNBC, we conducted immunoprecipitation (IP) assays coupled with mass spectrometry (MS) analysis of potential KLHL29-interacting proteins in BT549 and CAL51 cell lines (Fig. 4A). As a result, we identified a total of 46 proteins that potentially interacted with Flag-KLHL29 in BT549 cells, and 69 proteins in CAL51 cells (Fig. 4B). The DEAD-box helicase family member DDX3X was one of the top candidates, as this protein that plays a vital role in cancer progression was revealed in both cell lines (Fig. 4C). DDX3X was upregulated in TNBC tissues compared with corresponding normal tissues in FUSCC-TNBC proteomic database (Supplementary Fig. S3A) [12]. In addition, DDX3X protein expression levels were significantly higher in TNBC or basal-like breast cancer than those in other subtypes through CPTAC proteomic database (Supplementary Fig. S3B,S3C). Moreover, we performed IHC staining analysis and found that patients with higher DDX3X expression had worse prognoses than those with lower DDX3X expression levels (Supplementary Fig. S3D–S3F). Multivariate cox regression analysis showed that high DDX3X expression was an independent risk factor for decreased survival outcomes in patients with TNBC (Supplementary Tables S3 and S4). As expected, knockdown of DDX3X impaired TNBC cell proliferation (Supplementary Fig. S3G–S3L), migration, and invasion (Supplementary Fig. S3M–S3P), suggesting that DDX3X is an oncoprotein and KLHL29 might exert its tumor suppressive functions by inhibiting DDX3X, which is further investigated below.

To confirm the interaction between KLHL29 and DDX3X, we performed reciprocal co-IP assays to show that exogenous KLHL29 indeed binds to exogenous DDX3X in HEK293T cells (Fig. 4D, E). Also, their endogenous interaction was verified in MDA-MB-231 cells (Supplementary Fig. S4A). Considering KLHL29 contains six Kelch repeat motif, BTB/POZ domain, and BACK domain, we tested to which domain(s) DDX3X can bind by generating a series of KLHL29 deletion mutants (Fig. 4F). The result showed that DDX3X bound to the Kelch domain (M4, amino acids 585-875) of KLHL29, as demonstrated by the domain-mapping experiment (Fig. 4G). The immunofluorescence confocal microscopic analysis confirmed that KLHL29 was partially co-localized with DDX3X in cytoplasm in BT549 and CAL51 cells (Fig. 4H), again indicating the interaction between these two proteins.

We then investigated whether KLHL29 regulated DDX3X levels, as many KLHL members can serve as a component of the CUL3 E3-ligase complex [23, 24]. Western blotting analysis showed that ectopic expression of KLHL29 reduced DDX3X protein levels (Fig. 4I), whereas knockdown of KLHL29 elevated DDX3X protein levels (Fig. 4J). As expected, KLHL29 did not influence mRNA levels of DDX3X (Supplementary Fig. S4C–S4F), indicating that KLHL29 regulates DDX3X expression at the post-transcriptional level. We also determined the clinical correlation between KLHL29 and DDX3X by performing IHC staining analysis of their expression in TNBC specimens. Representative IHC images were shown in Fig. 4K, L. KLHL29 expression was negatively correlated with DDX3X expression (Fig. 4M), again suggesting that KLHL29 represses DDX3X expression in TNBC. Thus, we tested whether KLHL29 suppressed TNBC progression by repressing DDX3X. Consistent with the former results (Fig. 2), ectopic KLHL29 inhibited TNBC proliferation, migration, and invasion, whereas the overexpression of DDX3X markedly reversed these phenotypes (Supplementary Fig. S5A–S5H). Together, these results demonstrate that KLHL29 suppresses TNBC by interacting with and inhibiting the expression of DDX3X.

### KLHL29 promotes the proteasomal degradation of DDX3X

**Fig. 5:**
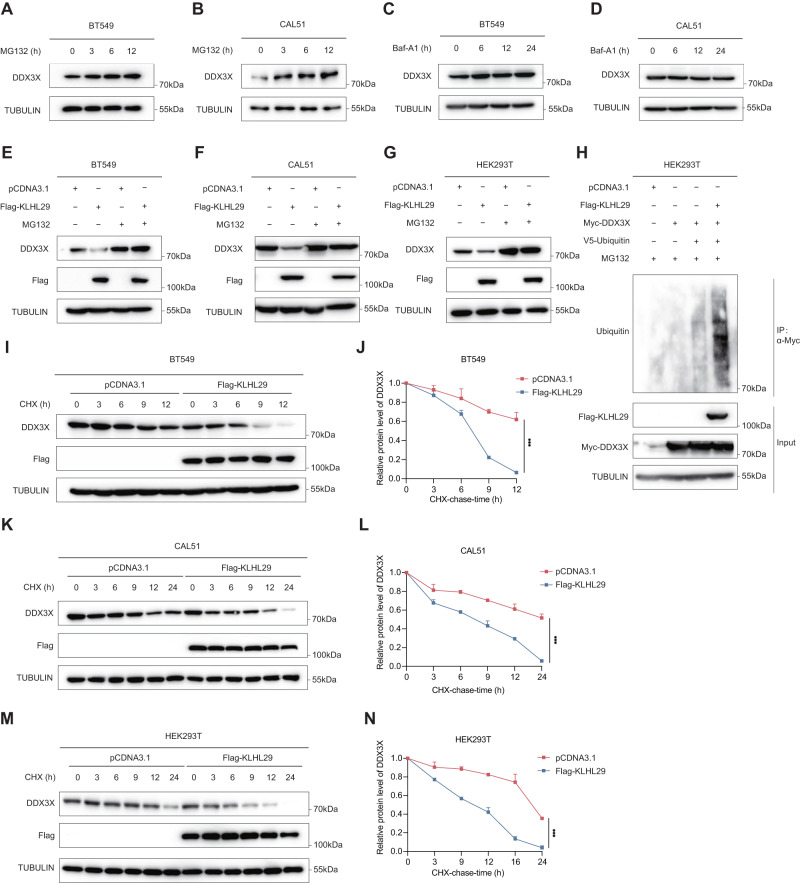
KLHL29 promotes the ubiquitin-proteasome degradation of DDX3X. **A**, **B** Protein levels of DDX3X in BT549 (**A**) and CAL51 (**B**) cells are elevated upon the treatment with 20 μM MG132 at the indicated time points. **C**, **D** Protein levels of DDX3X in BT549 (**C**) and CAL51 (**D**) cells are not affected by 200 nM Baf-A1 treatment at the indicated time points. **E**–**G** MG132 prevents KLHL29-mediated DDX3X degradation in BT549 (**E**), CAL51 (**F**) and HEK293T (**G**) cells. Cells expressing the empty vector or Flag-KLHL29 plasmid were treated with DMSO or 20 μM MG132 for 6 h before harvested for western blotting. **H** KLHL29 enhances the ubiquitination level of DDX3X. HEK293T cells were transfected with the indicated plasmids and treated with 20 μM MG132 for 6 h before harvested for ubiquitination assays. **I**–**N** The half-life of DDX3X is shortened upon KLHL29 overexpression in BT549 (**I**), CAL51 (**K**) and HEK293T (**M**) cells. Cells expressing the empty vector or Flag-KLHL29 plasmid were treated with 100 μg/mL of CHX and harvested for western blotting at the indicated time points. Quantification of relative KLHL29 protein levels (KLHL29/TUBULIN) by ImageJ are shown in **J**, **L**, and **N**. ***p < 0.001 by two-tailed Student’s *t* test.

Two major protein degradation systems in eukaryotic cells involve the ubiquitin-proteasome system and autophagy-lysosome system [25]. We first treated TNBC cells with the proteasome inhibitor MG132 or the autophagy inhibitor bafilomycinA (Baf-A1), and found that DDX3X levels increased in a time-dependent manner upon MG132, but not Baf-A1, treatment (Fig. 5A–D), suggesting that this protein is mainly subjected to the proteasomal degradation in TNBC cells. In addition, KLHL29-mediated DDX3X reduction was through proteasomal degradation, as MG132 could completely abrogate this reduction (Fig. 5E–G). Whereas, our results revealed that Baf-A1 was unable to restore the DDX3X levels following KLHL29 overexpression (Supplementary Fig. S6A, S6B), further demonstrating that KLHL29 induces DDX3X protein degradation through the ubiquitin-proteasome system rather than autophagy. We also showed that ectopic expression of KLHL29 enhanced the ubiquitination level of DDX3X (Fig. 5H). Moreover, we examined the half-life of DDX3X protein in response to KLHL29 overexpression by conducting cycloheximide (CHX)-chase assays. DDX3X protein half-life was drastically shortened by ectopic expression of KLHL29 in BT549 (Fig. 5I, J), CAL51 (Fig. 5K, L), and HEK293T (Fig. 5M, N) cells. These results demonstrate that KLHL29 promotes proteolytic degradation of DDX3X through the ubiquitin-proteasome system.

### CUL3 E3-ligase is required for KLHL29-mediated DDX3X degradation

**Fig. 6:**
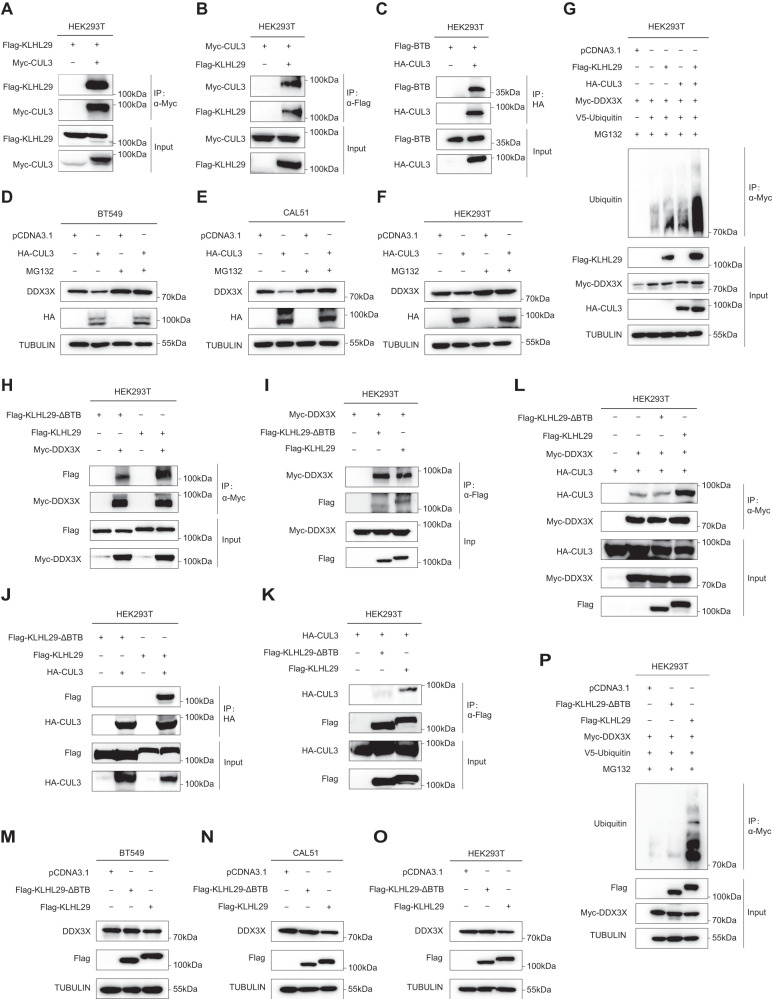
The BTB domain of KLHL29 recruits CUL3 E3-ligase to degrade DDX3X. **A**, **B** Exogenous KLHL29 interacts with exogenous CUL3. HEK293T cells were transfected with plasmids encoding Flag-KLHL29 and Myc-CUL3, followed by co-IP and western blotting assays. **C** CUL3 interacts with the BTB domain of KLHL29 in HEK293T cells. **D**–**F** Ectopic expression of CUL3 reduces DDX3X protein levels. MG132 blocks CUL3-mediated DDX3X degradation in BT549 (**D**), CAL51 (**E**) and HEK293T (**F**) cells. Cells expressing the empty vector or HA-CUL3 plasmid were treated with DMSO or 20 μM MG132 for 6 h before harvested for western blotting. **G** KLHL29 enhances CUL3-induced ubiquitination of DDX3X. **H**, **I** The BTB domain-deleted variant of KLHL29 (KLHL29-ΔBTB) interacts with DDX3X. HEK293T cells were transfected with plasmids encoding Myc-DDX3X and Flag-KLHL29-ΔBTB or Flag-KLHL29, followed by co-IP and western blotting assays. Flag-KLHL29 is set as control. **J**, **K** KLHL29-ΔBTB fails to bind to CUL3. HEK293T cells were transfected with plasmids encoding HA-CUL3 and Flag-KLHL29-ΔBTB or Flag-KLHL29, followed by co-IP and western blotting assays. Flag-KLHL29 is set as control. **L** Ectopic expression of KLHL29, but not KLHL29-ΔBTB, enhances the interaction of CUL3 and DDX3X. HEK293T cells were transfected with plasmids encoding Myc-DDX3X and HA-CUL3 together with Flag-KLHL29-ΔBTB or Flag-KLHL29, followed by co-IP and western blotting assays. **M**–**O** KLHL29-ΔBTB fails to regulate the protein levels of DDX3X in BT549 (**M**), CAL51 (**N**) and HEK293T (**O**) cells. **P** KLHL29-ΔBTB does not regulate the ubiquitination levels of DDX3X.

Several Kelch-like proteins can recruit the CUL3 E3 ubiquitin ligase via the BTB domain to the substrates for proteasomal degradation [23]. We investigated whether CUL3 is involved in KLHL29-mediated degradation of DDX3X. Reciprocal co-IP assays revealed that KLHL29 indeed interacts with CUL3 (Fig. 6A, B; Supplementary Fig. S6C, 6D) via the BTB domain (Fig. 6C and Supplementary Fig. S6E). Also, their endogenous interaction was verified in MDA-MB-231 cells (Supplementary Fig. S4B). Based on FUSCC-TNBC and TCGA RNA-seq dataset, CUL3 was significantly downregulated in TNBC tissues compared with adjacent normal tissues (Supplementary Fig. S6F–S6I). Ectopic expression of CUL3 promoted the proteasomal degradation of DDX3X, as MG132 treatment rescued this downregulation of DDX3X (Fig. 6D–F). In addition, the depletion of CUL3 could abolish the inhibitory effect of KLHL29 on DDX3X protein expression (Supplementary Fig. S6J, S6K). Moreover, ectopic CUL3 enhanced the ubiquitination levels of DDX3X, and this effect was strengthened through further overexpression of KLHL29 (Fig. 6G).

Next, we investigated the possible role of KLHL29’s BTB domain in the regulation of DDX3X by constructing a BTB domain-deleted variant (KLHL29-ΔBTB). Although KLHL29-ΔBTB still interacted with DDX3X (Fig. 6H, I), it failed to bind to CUL3 (Fig. 6J, K), suggesting that the BTB domain of KLHL29 is critical for the recruitment of CUL3 to DDX3X. Remarkably, ectopic KLHL29, but not KLHL29-ΔBTB, dramatically increased the interaction between CUL3 and DDX3X (Fig. 6L; Supplementary Fig. S6L), reduced DDX3X levels (Fig. 6M–O), and enhanced CUL3-induced DDX3X ubiquitination (Fig. 6P). Accordingly, ectopic KLHL29, but not KLHL29-ΔBTB, markedly suppressed proliferation (Supplementary Fig. S7A–S7D), migration, and invasion (Supplementary Fig. S7E–S7H) of TNBC cells. Together, these results demonstrate that KLHL29’s BTB domain mediates DDX3X degradation through the CUL3 E3-ligase to suppress TNBC progression.

### The KLHL29-DDX3X axis regulates cell cycle progression in triple-negative breast cancer

**Fig. 7:**
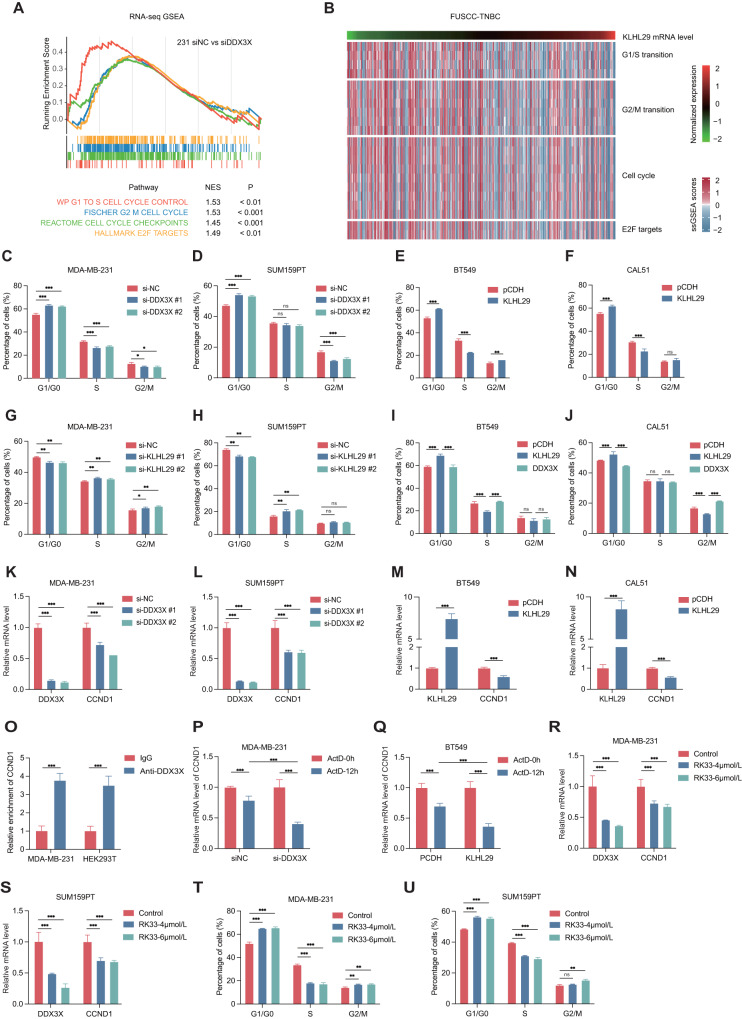
The KLHL29-DDX3X-axis regulates TNBC cell cycle progression. **A** Enrichment plot of cell cycle pathways after DDX3X knockdown in MDA-MB-231 cells by GSEA analysis. **B** The ssGSEA scores of cell cycle pathways according to KLHL29 mRNA levels based on FUSCC-TNBC dataset. **C**, **D** Depletion of DDX3X leads to an accumulation of cell cycle at G0/G1 phase in MDA-MB-231 (**C**) and SUM159PT (**D**) cells by flow cytometry analysis. Cells were stained with propidium iodide (PI) for the cell cycle assays. The percentage of cells in the G1/S/G2M phase are shown. **E**, **F** Ectopic expression of KLHL29 leads to an accumulation of cell cycle at G0/G1 phase in BT549 (**E**) and CAL51 (**F**) cells by flow cytometry analysis. **G**, **H** Knockdown of KLHL29 leads to a reduction of cell cycle at G0/G1 phase in MDA-MB-231 (**G**) and SUM159PT (**H**) cells by flow cytometry analysis. **I**, **J** The accumulation of cell cycle at G0/G1 phase induced by ectopic KLHL29 is restored by re-expression of DDX3X in BT549 (**I**) and CAL51 (**J**) cells by flow cytometry analysis. **K**, **L** Depletion of DDX3X represses the mRNA expression of CCND1 in MDA-MB-231 (**K**) and SUM159PT (**L**) cells by RT-qPCR analysis. **M**, **N** Ectopic expression of KLHL29 represses the mRNA expression of CCND1 in BT549 (**M**) and CAL51 (**N**) cells by RT-qPCR analysis. **O** DDX3X binds to the transcripts of CCND1. MDA-MB-231 and HEK293T cells were transfected with plasmids encoding Myc-DDX3X, followed by the RIP assays using anti-Myc antibody. **P** Depletion of DDX3X promotes the destabilization of CCND1 mRNA. ActD (5 μg/ml) was used to block RNA Pol II-mediated transcription in MDA-MB-231 cells. **Q** Ectopic expression of KLHL29 promotes the destabilization of CCND1 mRNA. **R**, **S** RK33 treatment reduces DDX3X and CCND1 mRNA level in MDA-MB-231 (**R**) and SUM159PT (**S**) cells by RT-qPCR analysis. **T**, **U** RK33 treatment leads to an accumulation of cell cycle at G0/G1 phase in MDA-MB-231 (**T**) and SUM159PT (**U**) cells by flow cytometry analysis. *p < 0.05, **p < 0.01, ***p < 0.001, ns, no significance by two-tailed Student’s t test.

Since DDX3X is an RNA-binding protein involved in the regulation of gene expression, including transcription, mRNP assembly, and pre-mRNA splicing [26, 27], we conducted transcriptomic analysis to explore the potential molecular mechanism behind the role of DDX3X in TNBC. Gene set enrichment analysis (GSEA) indicated that DDX3X was correlated with cell cycle-associated pathways in MDA-MB-231 and SUM159PT cells (Fig. 7A; Supplementary Fig. S8A). Kyoto Encyclopedia of Genes and Genomes (KEGG) pathway and Gene Ontology (GO) analyses also revealed that cell cycle-associated genes were mostly enriched in DDX3X-depleted cells (Supplementary Fig. S8B–S8D). In addition, through the single-sample gene set enrichment analysis (ssGSEA) based on the FUSCC-TNBC RNA-seq dataset, we also found that the expression levels of KLHL29 were correlated with the dysregulated expression of cell cycle-associated genes (Fig. 7B). These findings suggested that the KLHL29-DDX3X axis might regulate cell cycle progression in TNBC. To test this hypothesis, we conducted a series of flow cytometry analysis of cell cycle. Intriguingly, depletion of DDX3X triggered an accumulation of cell cycle at G0/G1 phase in MDA-MB-231 and SUM159PT cells (Fig. 7C, D). In line with these results, ectopic KLHL29 induced cell cycle arrest at G0/G1 phase (Fig. 7E, F), whereas KLHL29 knockdown promoted the G1/S transition (Fig. 7G, H). More intriguingly, ectopic KLHL29-induced cell cycle arrest could be restored by the re-expression of DDX3X (Fig. 7I, J), supporting an important role for the KLHL29-DDX3X axis in the regulation of cell cycle.

Mechanistically, depletion of DDX3X repressed the expression of CCND1, CCNE1, and CCNA2, genes encoding cyclins that promote the G1/S transition [28], as evidenced by our transcriptomic results and validated by RT-qPCR analysis (Fig. 7K, L; Supplementary Fig. S8E, S8F). Likewise, the overexpression of KLHL29 inhibited the expression of the three cyclin genes (Fig. 7M, N; Supplementary Fig. S8G, S8H). Interestingly, we found that DDX3X binds to the transcripts of CCND1 by RNA immunoprecipitation assay (Fig. 7O). This binding resulted in the stabilization of CCND1 mRNA, as depleting DDX3X or ectopic KLHL29 significantly reduced CCND1 mRNA levels when DNA transcription was blocked by 5 μg/ml Actinomycin D (ActD, Fig. 7P, Q). RK33, which serves as a small-molecule DDX3 inhibitor with radiosensitizing properties has been demonstrated to perform promising anticancer activity in different types of cancer including lung cancer, breast cancer and sarcoma [29–31]. Our results confirmed that RK33 dramatically reduces both mRNA and protein levels of DDX3X and thus the expression of CCND1 in TNBC cells (Fig. 7R, S; Supplementary Fig. S8I, S8J). The flow cytometry analysis showed that RK33 treatment, like DDX3X knockdown, significantly induces cell cycle arrest at G0/G1 phase (Fig. 7T, U). Therefore, these results demonstrate that the KLHL29-DDX3X axis regulates cell cycle progression by controlling the expression of cell cycle-associated genes.

### Combining the DDX3X inhibitor RK33 with platinum-based chemotherapy effectively suppresses triple-negative breast cancer in vitro and in vivo

Platinum-based chemotherapy has been used for the treatment of TNBC, while the G1/S cell cycle checkpoint is one of the main mechanisms that control cellular response to DNA damage and chemosensitivity [32, 33]. We sought to investigate if interference with DDX3X function improves TNBC sensitivity to platinum agents by modulating the G1/S transition. First, we evaluated the anti-cancer efficacy of RK33 in TNBC cells. As shown in Supplementary Fig. S9A–S9D, DDX3X depletion or KLHL29 overexpression markedly increased half-maximal inhibitory concentration (IC50) values of RK33. Consistently, TNBC patient-derived organoids sustaining higher levels of DDX3X (Supplementary Fig. S9E, S9F) or lower levels of KLHL29 (Supplementary Fig. S9G, S9H) exhibited higher sensitivity to RK33. Therefore, TNBC that usually expresses high levels of DDX3X is susceptible to RK33-induced cytotoxicity.

**Fig. 8:**
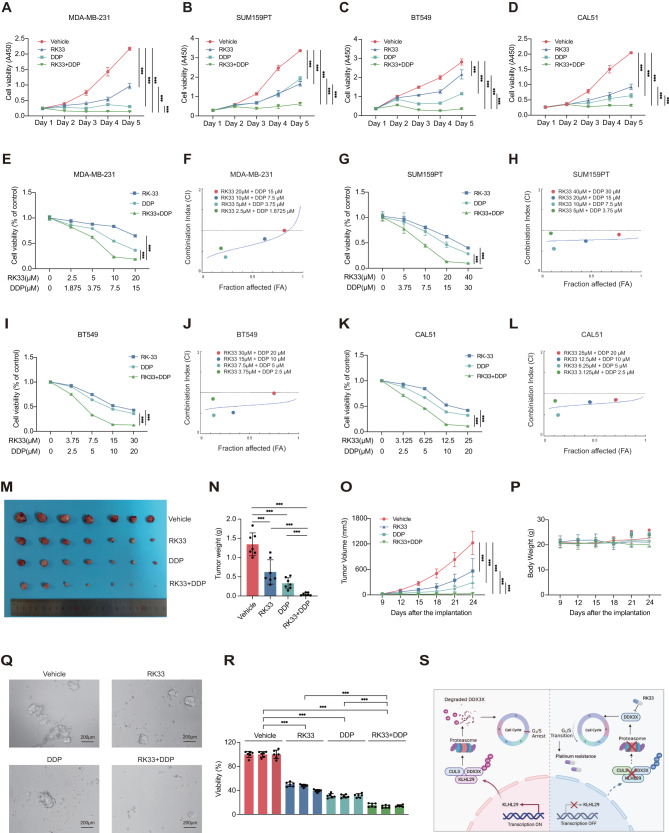
The combination of DDX3X inhibitor RK33 with cisplatin suppresses TNBC progression in vitro and in vivo. **A**–**D** Treatment with RK33 or cisplatin alone reduces cell growth of MDA-MB-231 (**A**), SUM159PT (**B**), BT549 (**C**), and CAL51 (**D**) cells compared with vehicle treatment by cell viability assays using the Cell Counting Kit-8. The combination of RK33 and cisplatin more dramatically impairs TNBC cell growth compared with each of the individual treatments. **E**, **F** RK33 and cisplatin synergistically suppress the growth of MDA-MB-231 cells. Cells were treated with combinations of agents, followed by the cell viability assays (**E**) and the Chou-Talalay analysis (**F**). **G**, **H** RK33 and cisplatin synergistically suppress the growth of SUM159PT cells. **I**, **J** RK33 and cisplatin synergistically suppress the growth of BT549 cells. **K**, **L** RK33 and cisplatin synergistically suppress the growth of CAL51 cells. **M**–**P** The combination of RK33 and cisplatin dramatically suppresses the growth of MDA-MB-231 cell-derived orthotopic xenograft tumors in BALB/c female nude mice (*n* = 7 mice per group). Representative tumor images (**M**), tumor growth rate (**N**), tumor weight (**O**) and body weight (**P**) are shown. **Q**, **R** The combination of RK33 and cisplatin dramatically suppresses the growth of organoids derived from TNBC patients. Representative images of organoids after drug treatment (**Q**), and organoid cell viability evaluated by CellTiter-Glo 3D Cell viability assay (**R**) are shown. **S** The proposed working model. KLHL29 recruits the CUL3 E3-ligase to promote ubiquitination and proteasomal degradation of DDX3X, leading to the cell cycle arrest at G0/G1 phase. Targeting DDX3X by the small molecule RK33 sensitizes TNBC to platinum-based treatment.

Next, we evaluated the combination effects of RK33 and platinum agents on TNBC cell growth. Cell viability assays were performed to show that the combination of RK33 with cisplatin or carboplatin more drastically inhibits TNBC cell growth than does each of the individual treatments (Fig. 8A–D; Supplementary Fig. S10A–S10D). We then investigated whether RK33 and platinum agents synergistically inhibit TNBC by calculating the combination index (CI). The combinations of RK33 with cisplatin at different concentrations displayed synergistic effects with the values of CI < 1 in multiple TNBC cell lines (Fig. 8E–L). Consistently, combining RK33 with carboplatin also showed a synergistic anti-tumor effect (Supplementary Fig. S10E–S10L).

To translate the cell-based results above into clinical relevance, we tested the combination effects of RK33 with cisplatin or carboplatin in xenograft mouse models and PDO models. BALB/c-nude mice were inoculated with MDA-MB-231 cells orthotopically, and randomly divided into four groups for different treatments, including vehicle, RK33, platinum agents, and the combination of RK33 with platinum. While the single-agent treatment significantly inhibited tumor growth in vivo, the combinational treatment suppressed the tumor growth to a greater extent, as evidenced by the tumor size, weight, and growth rate (Fig. 8M–O, Supplementary Fig. S10M–S10O). The side effects of the treatments were tolerable, because the average weight of treated mice was comparable to that of control mice (Fig. 8P; Supplementary Fig. S10P). In addition, the anti-TNBC efficacy of the combination therapy was further validated using PDO models (Fig. 8Q, R, Supplementary Fig. S10Q, S10R). Taken together, these results demonstrate that the combination of RK33 and platinum-based chemotherapy can effectively suppress TNBC in vitro and in vivo.

To further validate our results that RK33 sensitizes TNBC cells to cisplatin by modulating the G1/S transition, cell viability assays were performed to show that the combination of RK33 with paclitaxel could more dramatically inhibit TNBC cell growth than RK33 single-agent treatment (Supplementary Fig. S11A, S11B). We then investigated whether RK33 and paclitaxel synergistically inhibit TNBC by calculating the CI. The combinations of RK33 with paclitaxel at different concentrations displayed no synergistic effects with the values of CI > 1 in multiple TNBC cell lines (Supplementary Fig. S11C–S11F). These findings again suggest that the G1/S cell cycle regulation is crucial to the drug combination with RK33.

## Discussion

The molecular basis of the progression and chemoresistance of TNBC involves complex networks of gene interactions. Here, we uncovered the role for the KLHL29-DDX3X signaling cascade in the regulation of TNBC development and therapy. KLHL29 is downregulated in TNBC tissues compared with the adjacent normal tissues, and low expression levels of KLHL29 in tumors are significantly associated with unfavorable prognoses (Fig. 1). Ectopic KLHL29 suppresses, while depleting KLHL29 promotes, the growth and metastasis of TNBC (Figs. 2 and 3; Supplementary Figs. S1 and S2). Acting as an adapter that bridges the E3-ligase CUL3 and the substrate DDX3X, KLHL29 executes the tumor suppressive function by enhancing the proteasomal degradation of DDX3X, consequently leading to the downregulation of cyclins and cell cycle arrest at G0/G1 phase (Figs. 4–7; Supplementary Figs. S3–S8). Since TNBC generally expresses low levels of KLHL29 and high levels of DDX3X, we further demonstrate that combining the DDX3X inhibitor RK33 with platinum agents synergistically suppresses TNBC growth in vitro and in vivo (Fig. 8; Supplementary Figs. S9–S11).

KLHL29 belongs to the KLHL gene superfamily, which generally contains a BTB/POZ domain, a BACK domain, and six Kelch repeat motifs []. KLHL proteins usually participate in various critical cellular processes by regulating the function of substrate proteins. In the CUL3-KLHL ubiquitin ligase complex, the BTB domain binds to CUL3, while the Kelch domain is responsible for the recruitment of specific substrates [23]. Through a MS analysis coupled with a series of co-IP assays, we identified the RNA-binding protein DDX3X as a novel binding partner of KLHL29 (Fig. 4A–H). Our study further indicated that KLHL29 recruits CUL3 to promote the ubiquitination and proteasomal degradation of DDX3X, which was supported by several lines of evidence (Figs. 4–6). First, KLHL29 interacted with CUL3 in TNBC and HEK293T cells (Fig. 6A–C; Supplementary Fig. S6C–6E). Second, KLHL29 increased the interaction between CUL3 and DDX3X (Fig. 6L; Supplementary Fig. S6L). Third, CUL3 induced the ubiquitination and proteasomal degradation of DDX3X, which could be further enhanced by ectopic KLHL29 (Fig. 6D–G). Fourth, the BTB domain of KLHL29 was critical for the recruitment of CUL3, as KLHL29-ΔBTB that lacked the BTB domain failed to bind to CUL3, enhance CUL3-DDX3X interaction, and promote DDX3X ubiquitination and degradation (Fig. 6H–P). Finally, the expression levels of KLHL29 were negatively correlated with DDX3X expression in TNBC samples (Fig. 4K–M). Therefore, our study for the first time demonstrates the tumor suppressive role and the underlying mechanism for KLHL29 in TNBC.

KLHL29-binding partner, DDX3X, is a member of the DEAD (Asp-Glu-Ala-Asp)-box helicase family [34]. The DEAD-box helicase family is the largest group of RNA helicase family in eukaryotes, participating in all stages of RNA metabolism [35–39]. Recent evidence highlights the important functions of DDX3X in tumorigenesis and cancer progression, although it may play a tumor suppressive or an oncogenic role depending on the context of different cancers [27, 40–42]. Our study showed that DDX3X is upregulated in TNBC samples compared to adjacent normal breast tissues, and high levels of DDX3X are associated with poor survival outcomes (Supplementary Fig. S3A–S3F). In addition, DDX3X served as an oncogenic protein in TNBC, as the overexpression of DDX3X completely restored KLHL29-mediated inhibition of TNBC (Supplementary Fig. S3G–S3P, S5A–S5H). The tumor-promoting effect of DDX3X was partially attributed to its ability to regulate the expression of multiple cell cycle-associated genes and thus promote the G1/S transition (Fig. 7C–N; Supplementary Fig. S8A–S8H). Specifically, we showed that DDX3X binds to CCND1 mRNA and induces its stabilization (Fig. 7O–Q). More importantly, DDX3X knockdown or treatment of cells with the DDX3X inhibitor RK33 significantly triggered cell cycle arrest at G0/G1 phase (Fig. 7C–J, T, U). These findings indicate that DDX3X could be a potential therapeutic target in TNBC.

Platinum-based chemotherapy has been used for the treatment of TNBC in the neoadjuvant and metastatic settings [43–45]. Cell cycle regulation is regarded as one of the major determinants for cancer sensitivity to platinum agents [32, 33, 46]. Also, the amplification or overexpression of cyclin genes, such as CCND1 and CCNE1, is associated with platinum resistance in various cancers [47–49]. Interestingly, our previous study revealed that CCND1 and CCNE1 are amplified or overexpressed in around 50% of TNBC patient samples [27]. It was therefore reasonable to test whether inhibition of DDX3X and thus cyclin gene expression leads to chemosensitization of TNBC to platinum agents. Indeed, our results further demonstrated that combining RK33 with cisplatin or carboplatin can synergistically or coordinately suppress TNBC growth using in vitro, mouse xenograft, and PDO models (Fig. 8; Supplementary Fig. S10). Altogether, these findings provide a promising and alternative combinational therapy for TNBC that sustains low expression of KLHL29 and high expression of DDX3X and cyclins.

In conclusion, our study uncovers KLHL29 as a tumor suppressor in TNBC. KLHL29 recruits the CUL3 E3-ligase to promote ubiquitination and proteasomal degradation of DDX3X, resulting in the repression of cyclin genes and cell cycle arrest. TNBC expresses low levels of KLHL29 and high levels of DDX3X. Thus, targeting DDX3X by the small molecule RK33 sensitizes TNBC to platinum-based treatment by arresting cell cycle at G0/G1 phase, which can be exploited to develop a novel combinational therapy for TNBC.

## Materials and methods

### Clinical samples and datasets

TNBC specimens were obtained from patients who underwent surgery at the Department of Breast Surgery in the First Hospital of China Medical University without any therapy before surgery. All patients were diagnosed clearly by pathologists with complete follow-up data. The experimental procedures were conducted in accordance with the Declaration of Helsinki and were approved by the Institutional Ethics Review Board of the First Hospital of China Medical University. Written informed consent was obtained from all patients. The FUSCC-TNBC cohort, the Cancer Genome Atlas (TCGA) database, and the Clinical Proteomic Tumor Analysis Consortium (CPTAC) database were also analyzed.

### Mouse xenograft experiments

4 to 6 weeks old female NOD/SCID and BALB/c-nude mice were purchased from the Department of Laboratory Animal Science in Shanghai Medical College of Fudan University. To evaluate the effect of KLHL29 overexpression on tumor growth, 1 × 10^6^ MDA-MB-231 cells that overexpress KLHL29 or an empty vector (pCDH-CMV-MCS-EF1-puro) were harvested and resuspended in a 100 μl volume (PBS: Matrigel = 1:1) and were injected orthotopically into the mammary fat pads of NOD/SCID mice. To evaluate the effect of KLHL29 knockdown on tumor growth, 1 × 10^6^ MDA-MB-231 cells stably expressing shNC or sh-KLHL29 were injected orthotopically into the mammary fat pads of NOD/SCID mice. The combination effects of RK33 with cisplatin or carboplatin were tested in xenograft mouse models. BALB/c-nude mice were inoculated with MDA-MB-231 cells orthotopically, and randomly divided into four groups for different treatments, including vehicle, RK33, platinum agents, and the combination of both. RK33 (20 mg/kg) or cisplatin (3 mg/kg) in a volume of 200 μl volume was administered intraperitoneally three times a week. RK33 (20 mg/kg) or carboplatin (15 mg/kg) in a volume of 200 μl volume was administered intraperitoneally three times a week. The investigator was blinded to the group allocation when the inoculation was conducted. Tumor growth was monitored with electronic digital calipers in two dimensions. Tumor volume was calculated according to the formula: volume = length × width × width × 0.52. Mice were killed by euthanasia and tumors were harvested for future analyses. The animal protocols were in accordance with ethical guidelines and approved by the Animal Welfare Committee of Shanghai Medical College at Fudan University.

### Organoid

Patient-derived organoids (PDOs) were derived from post-surgery specimens of TNBC patients and cultured based on previously described methods [50]. Fresh breast cancer tissues were cut into 1–3 mm^3^ pieces and were digested with collagenase and hyaluronidase in digestion buffer on an orbital shaker for 1–2 h at 37 °C. The digested tissue suspension was passed through a 100 μm filter, centrifuged at 1500 rpm for 5 min at room temperature and resuspended in 10 mL of TAC buffer incubating for 3 min to remove red pellet. Dissociated cell clusters were centrifuged 5 min at 1500 rpm and resuspended in digestion buffer and spun down again. Cell clusters were resuspended in Basement Membrane Extract (BME) type-2 (Trevigen, 3533-010-02) and 40 mL drops of BME-cell suspension were solidified on prewarmed 24-well suspension culture plates at 37 °C for 20 min. The organoids were cultured in breast cancer organoid medium and maintained at 37 °C in a 5% CO_2_-humidified atmosphere. After 3–5 passages, the organoids were plated into 384-well plate, and incubated for another 5 days before being subjected to drug treatments. Organoids with drugs were cultured for 1 week before testing the viability. Organoid cell viability was evaluated with a CellTiter-Glo 3D Cell viability assay (Promega, #G9683) after treatment with the indicated drugs. On the last day, photos were taken to observe changes in organoids after drug treatment.

### Statistical analysis

Statistical analyses were carried out using GraphPad (version 9.4.1), R software (version 4.0.3) and SPSS (version 26.0). Statistical analyses of two groups were calculated using Wilcoxon rank-sum tests, two-tailed Student’s t tests or two-way ANOVA tests. Correlation coefficients were calculated using the Spearman test. The survival curves were generated by the Kaplan-Meier plot method and analyzed by the log-rank test. Multivariate Cox proportional hazard models provided hazard ratios with 95% confidence intervals. Data was presented as the mean ± standard deviation (SD) from at least three independent experiments. *P* < 0.05 was considered as statistically significant (*p < 0.05; **p < 0.01; ***p < 0.001; ns, no significance).

### Supplementary information

Supplementary file

## Data Availability

All study data are included in the article and/or Supplementary materials.
