# Supplementary material for: KLHL29-mediated DDX3X degradation promotes chemosensitivity by abrogating cell cycle checkpoint in triple-negative breast cancer
Source: Oncogene. 2023 Oct 16;42(47):3514–28. doi: 10.1038/s41388-023-02858-5 (PMC10656286; doi:10.1038/s41388-023-02858-5)
Supplement: Supplementary file 1 — Supplementary file [file 41388_2023_2858_MOESM1_ESM.pdf]

Supplementary information

KLHL29-mediated DDX3X degradation promotes chemosensitivity by abrogating cell cycle checkpoint in triple-negative breast cancer.

Litong Yao[1], Qian Hao[2], Mozhi Wang[1], Yuhai Chen[1], Hongyi Cao[3], Qiang Zhang[4], Keda Yu[2], Yizhou Jiang[2], Zhiming Shao[2], Xiang Zhou[2,5,#], Yingying Xu[1,#]

1. Department of Breast Surgery, the First Hospital of China Medical University, Shenyang, Liaoning, China.

2. Fudan University Shanghai Cancer Center, Fudan University, Shanghai, China

3. Department of Pathology, The First Hospital of China Medical University and College of Basic Medical Sciences, Shenyang, Liaoning, China

4. Department of Breast Surgery, Cancer Hospital of China Medical University, Liaoning Cancer Hospital & Institute, Shenyang, Liaoning, China

5. Shanghai Key Laboratory of Medical Epigenetics, International Co-laboratory of Medical Epigenetics and Metabolism (Ministry of Science and Technology), Institutes of Biomedical Sciences, Fudan University, Shanghai, China

# Corresponding Author:

Yingying Xu, E-mail: xuyingying@cmu.edu.cn

Xiang Zhou, Email: xiangzhou@fudan.edu.cn

This supplementary file includes:

Supplementary Materials and Methods

Supplementary Figure Legends

Supplementary Figures S1 to S11

Supplementary Tables S1 to S8

Supplementary Materials and Methods

Cell lines

Human TNBC cell lines (BT549, CAL51, SUM159PT, MDA-MB-231, LM2-4175, Hs578T, and HCC1806), and human embryonic kidney cell line (HEK293T) were stored in laboratory. These cells were cultured following the instructions from ATCC. All cell lines were routinely tested negative for mycoplasma contamination with a mycoplasma detecting kit (Yeasen, Shanghai, China) and were maintained at 37 °C in a 5% CO_2-humidified atmosphere.

Plasmids, siRNA construction and transfection

The plasmid expressing KLHL29 was purchased from Vigene Biosciences Inc. (Shandong, China). The corresponding cDNAs were amplified by PCR and cloned into the pCDH-CMV-MCS-EF1-puro vector and pCDNA3.1/Flag vector. The Myc-tagged plasmids expressing DDX3X and the HA-tagged plasmids expressing CUL3 were purchased from Sino Biological Inc. (Beijing, China). The Myc-tagged CUL3 plasmids were generated by inserting the full-length cDNA into the pCDNA3.1/Myc-His vector. The Flag-tagged plasmids expressing KLHL29 fragments (M1, amino acids 1 to 328; M2, amino acids 1 to 401; M3, amino acids 329 to 584; and M4, amino acids 585 to 875) were generated by the same approach using specific primers. The site-directed mutations in the Flag-tagged KLHL29 plasmids were generated by KOD-Plus Mutagenesis Kit (Toyobo, Japan).

The short hairpin (shRNA) oligos were annealed and cloned into the pLKO.1-Puro vector. The plasmids expressing shRNAs were designed by Sigma-Aldrich (USA). Plasmids transfection was conducted using PEI transfection reagent following the manufacturer's protocol (Polysciences, USA). siRNAs targeting KLHL29 and DDX3X were synthesized and purified by RiboBio (Guangzhou, China) and GenePharma (Shanghai, China). siRNA transfection was conducted using Lipofectamine 3000 transfection reagent according to the manufacturer's instructions (Thermo Fisher Scientific, USA). The related sequences were listed in Supplementary Table S5-S6.

Generation of lentiviral particles

The lentiviral expression vectors were introduced into HEK293T cells together with packing plasmids (psPAX2 and pMD2.G) to generate lentiviruses. The virus containing supernatant was collected 48 hours after transfection using and then used for cell infection. The cleared supernatant was filtered through a 0.45μm filter, aliquoted and stored at −80°C. Target cells were infected with retrovirus supernatant in the presence of 8 μg/ml polybrene (Santa Cruz Biotechnology, USA) and were selected using 1 μg/ml puromycin (MedChemExpress, China) for 1 weeks. The knockdown or overexpression efficiency was validated with qRT-PCR or western blotting.

RNA isolation and qRT-PCR

Total RNA was extracted using RNAiso Plus reagent (Takara, Japan) and reversely

transcribed into cDNA using the PrimeScript RT reagent Kit (Takara, Japan) according to the manufacturer's protocol. qRT-PCR was performed using SYBR Premix Ex Taq (Takara, Japan) on a QuantStudio 6 Flex Real-Time PCR System (Applied Biosystems, USA). The mRNA expression levels of genes were estimated using $2^{-\Delta\Delta CT}$ method and were shown relative to the expression levels of the reference gene. The primers sequences used for qRT-PCR were listed in Supplementary Table S7.

Western blotting

For western blotting, cultured cells were washed with phosphate-buffered saline (PBS) and lysed in lysis buffer consisting of 50 mM Tris/HCl (pH 7.5), 0.5% Nonidet P-40 (NP-40), 1 mM ethylene diamine tetraacetic acid (EDTA), 150 mM NaCl, 1 mM dithiothreitol, 0.2 mM phenylmethylsulfonyl fluoride, 10 mM pepstatin A, and 1 mM leupeptin. The protein concentration was determined using a BCA protein assay kit (Yeasen, Shanghai, China) and equal quantities of protein samples (10 to 100 mg) were used for western blotting analysis and resolved by SDS-PAGE. The separated proteins were transferred to polyvinylidene difluorie (PVDF) membranes (Merck Millipore, USA). The membranes were incubated with indicated primary antibody followed by an horseradish peroxidase (HRP)-conjugated secondary antibody after blocking the membrane with 5% non-fat milk (Sangon, Shanghai, China), and then detected by enhanced chemiluminescence detection kit (Yeasen, Shanghai, China). The details for antibody used in study were listed in Supplementary Table S8.

Immunoprecipitation

For the endogenous immunoprecipitation (IP) assay, proteins of 1 mg were incubated with the indicated antibodies or control IgG in a rotating incubator at 4 °C overnight and were then incubated with protein A or G beads (Santa Cruz Biotechnology, USA) for additional 1 to 2 hours. For the exogenous IP assay, proteins of 500 μg were incubated with indicated magnetic beads (Bimake, China) in a rotating incubator at 4 °C for 3 hours. The immunoprecipitates were washed at least three times with lysis buffer and were detected by western blotting with antibodies as indicated.

RNA immunoprecipitation Assay

For RNA immunoprecipitation (RIP) assay, cultured cells were washed with PBS and lysed in lysis buffer consisting of the protease inhibitor and RNase inhibitor. The supernatant was collected by centrifugation for 15 min at 14 500 rpm at 4 °C, and was incubated with indicated magnetic beads in a rotating incubator at 4 °C overnight. The immunoprecipitates were washed at least three times with lysis buffer containing the RNase inhibitor. The bound RNA was extracted by RNAiso Plus reagent and analyzed by RT-qPCR.

Cell viability and colony formation assays

Cells were counted after digestion and planted at a density of 2×10^3 in 96-well plates after transfection for 12 hours. Cell Counting Kit-8 (CCK-8) (Yeasen, Shanghai, China) was used for the determination of cell viability following the manufacturer's protocol.

The absorbance values at a wavelength of 450 nm were measured at the indicated times and used to plot the cell proliferation curve. For assessment of half-maximal inhibitory concentration (IC50), cell suspensions were seeded at a density of 6×10^3 cells in 96-well plates to determine the concentration that causes 50% inhibition of cell viability. Cells were treated with RK33 at serial and dilutions, the cell viability was measured by CCK-8 after culturing 24 to 48 hours. Colony formation assays were performed in a six-well plate in triplicate at a density of 1×10^3. The medium was changed every 3 days until the colonies were visible. Cells were fixed with methanol and stained with crystal violet after 14 days of culture.

Transwell migration and invasion Assays

For the cell migration and invasion assays, a total of 4-10 $\times 10^4$ cells were suspended in serum-free medium and planted in the top chambers (FALCON, USA). The top chambers were coated with Matrigel (BD Biosciences) for the invasion assay, coated without Matrigel for the migration assay. The lower chambers were covered with medium containing 20% FBS. After incubation for 12 to 48 hours, migrated and invaded cells on lower surface were fixed with methanol and stained with 0.1% crystal violet, and the unmigrated and uninvaded cells were removed using cotton swabs. The numbers of migrated and invaded cells were counted in randomly selected microscope fields and averaged.

Flow cytometry analysis

For the cell apoptosis assay, cells were collected, resuspended in binding buffer, and stained with Annexin V and 7-amino-actinomycin D (7-AAD) staining solution from the Annexin V-PE/7-AAD Apoptosis Detection Kit (Vazyme, Nanjing, China) according to the manufacturer's instructions. For the cell cycle assay, cells were fixed with precooled 70% ethanol overnight and incubated with propidium iodide (PI, MedChemExpress, China) staining buffer containing 50 μg/ml PI, 200 μg/ml RNase A, 0.1% Triton X-100 in PBS at 37°C for 30 min. The flow cytometry data were generated using a FC500 MPL flow cytometer (Beckham coulter, Indianapolis, IN, USA).

Immunohistochemistry (IHC)

Paraffin-embedded tissue sections were deparaffinized at 60 °C for 20 minutes and then subjected to xylene and a graded alcohol series. For IHC staining, the slides were heated with citrate or EDTA antigen retrieval solution for antigen unmasking, blocked with blocking reagents containing 2% goat serum, 2% BSA, and 0.05% Tween-20 in PBS at room temperature for 10 minutes, and then incubated with primary antibodies at 4°C overnight. Then, the sections were incubated with HRP-conjugated secondary antibody, developed in DAB reagents, counterstained with hematoxylin, dehydrated with a graded alcohol series, and mounted with coverslips and mounting medium. The IHC score was calculated by intensity of staining (0 = negative, 1 = weak, 2 = moderate, 3 = strong) and percentage of positive tumor cells staining (0 = negative, 1 ≤ 10%, 2 = 10–50%, 3 ≥ 50%), and an IHC score was calculated by multiplying the positive staining score by the staining intensity score. Interpretation of the IHC results was

performed by two independent pathologists.

Immunofluorescence staining and confocal assays

Cells were planted on the disc in 24-well plates and were fixed with methanol in −20°C overnight. The fixed cells were washed by PBS and blocked with 8% bovine serum albumin (BSA) in PBS for 1 hour followed by incubation with primary antibodies in 4 °C overnight. The cells were then washed and incubated with the corresponding secondary fluorescent antibodies. Cells were then placed on slides and stained with DAPI solution (Sigma-Aldrich, USA). Images were taken with a Leica fluorescence confocal microscope.

Transcriptome sequencing

RNA was extracted from MDA-MB-231 and SUM159PT cell lines treated with control siRNAs or three independent DDX3X-targeted siRNAs using RNAiso Plus reagent according to the manufacturer's protocol. RNA purity and quantification were conducted using the NanoDrop 2000 spectrophotometer (Thermo Scientific, USA). RNA integrity was evaluated using the Agilent 2100 Bioanalyzer (Agilent Technologies, USA). RNA sequencing libraries were constructed using TruSeq Stranded mRNA LT Sample Prep Kit (Illumina, USA). The transcriptome sequencing was performed by OE Biotech Co., Ltd. (Shanghai, China).

Mass spectrometry (MS) analysis

To analyze proteins that interact with KLHL29, CAL51 and BT549 cells stably expressing pCDH and Flag-KLHL29 were lysed in lysis buffer. Equal quantities of protein samples were subjected to IP assays using an anti-Flag antibody, and affinity-isolated KLHL29 protein was electrophoresed on SDS-PAGE. The gel was stained with Coomassie Brilliant Blue and was subjected to trypsin digestion and mass spectrometry (MS) analysis. The identification of proteins was processed using Proteome Discoverer 2.4, and identified peptides were validated utilizing a Percolator algorithm.

Chemicals

The following chemicals were used: RK33, a potent DDX3X inhibitor (Selleck); cisplatin, a chemotherapeutic drug (MedChemExpress); carboplatin, a chemotherapeutic drug (MedChemExpress); MG132, a potent proteasome inhibitor (Sigma-Aldrich); Cycloheximide, (CHX), a eukaryote protein synthesis inhibitor (Sigma-Aldrich); bafilomycinA, (Baf-A1), an autophagy inhibitor (Selleck); puromycin (MedChemExpress); Actinomycin D, (ActD), an mRNA synthesis inhibitor (MedChemExpress). Ampicillin and Kanamycin were obtained from Sangon Biotech.

Supplementary Figure Legends

Supplementary Fig. S1

A. Western blotting analysis of KLHL29 protein expression levels in six pairs of TNBC tissues and matched adjacent normal breast tissues. T, tumor; N, normal.

B and C. KLHL29 expression was confirmed in MDA-MB-231 cells stably expressing the empty vector pCDH-Flag and pCDH-Flag-KLHL29 by western blotting (A) and RT-qPCR (B) analyses.

D. Ectopic expression of KLHL29 suppresses cell proliferation of MDA-MB-231 cells by cell viability assays using the Cell Counting Kit-8.

E. Comparison of immunohistochemical staining scores of Ki67 among pCDH and KLHL29 group in xenograft tumors. Quantitative results (left) and representative images (right) are shown. Data are represented as mean ± SD. Wilcoxon rank-sum test.

F and G. KLHL29 expression was confirmed in MDA-MB-231 cells transfected with two independent shRNAs targeting KLHL29 by western blotting (D) and RT-qPCR (E) analyses.

H. Ablation of KLHL29 enhances cell proliferation of MDA-MB-231 cells by cell viability assays using the Cell Counting Kit-8.

I. Comparison of immunohistochemical staining scores of Ki67 among shNC and shKLHL29 group in xenograft tumors. Quantitative results (left) and representative images (right) are shown. Data are represented as mean ± SD. Wilcoxon rank-sum test.

***, $p < 0.001$ by two-tailed Student's t test or two-way ANOVA test.

Supplementary Fig. S2

A. Knockdown and re-expression of KLHL29 in MDA-MB-231 cells was confirmed by Western blotting analysis.

B. The promotion of cell proliferation induced by KLHL29 knockdown in MDA-MB-231 cells is restored by re-expression of KLHL29.

C and D. The promotion of cell migration and invasion induced by KLHL29 knockdown are restored by re-expression of KLHL29. Representative images of migrated and invaded cells (C), and corresponding quantitative results (D) are shown.

***, $p < 0.001$ by two-tailed Student's t test or two-way ANOVA test.

Supplementary Fig. S3

A. DDX3X protein expression is upregulated in TNBC tissues (n = 84) compared with adjacent normal breast tissues (n = 69) in FUSCC-TNBC proteomic dataset.

B. DDX3X protein expression is higher in TNBC than those in other subtypes through CPTAC proteomic database.

C. DDX3X protein expression is higher in basal-like breast cancer than those in other subtypes through CPTAC proteomic database.

D. Immunohistochemical staining analysis of DDX3X expression in TNBC specimens. Representative images are shown.

E and F. High expression level of DDX3X is associated with poor overall survival (E) and relapse-free survival (F). Log-rank test.

G and H. DDX3X expression was confirmed in MDA-MB-231 (G) and SUM159PT (H) cells transfected with two independent siRNAs targeting KLHL29 by western blotting analysis.

I and J. DDX3X expression was confirmed in MDA-MB-231 (I) and SUM159PT (J) cells transfected with two independent siRNAs targeting KLHL29 by RT-qPCR analysis.

K and L. Ablation of DDX3X impairs cell proliferation of MDA-MB-231 (K) and SUM159PT (L) cells by cell viability assays using the Cell Counting Kit-8.

M-P. Ablation of DDX3X suppresses cell migration and invasion of MDA-MB-231 and SUM159PT cells by cell migration and invasion assays using transwell chambers coated without and with Matrigel, respectively. Representative images of migrated and

invaded cells (M and N), and corresponding quantitative results (O and P) are shown.

***, $p < 0.001$ by two-tailed Student's t test or two-way ANOVA test.

Supplementary Fig. S4

A. Endogenous interaction between KLHL29 and DDX3X in MDA-MB-231 cells.

B. Endogenous interaction between KLHL29 and CUL3 in MDA-MB-231 cells.

C and D. RT-qPCR analysis of the mRNA expression levels of DDX3X in BT549 (C) and CAL51 (D) cells with KLHL29 overexpression.

E and F. RT-qPCR analysis of the mRNA expression levels of DDX3X in MDA-MB-231 (E) and SUM159PT (F) cells with KLHL29 knockdown.

***, $p < 0.001$, ns, no significance by two-tailed Student's t test.

Supplementary Fig. S5

A and B. Western blotting analysis showing the protein levels of Flag-KLHL29 and Myc-DDX3X in BT549 (A) and CAL51 (B) cells with or without KLHL29 overexpression and followed by transfection with empty vector or Myc-DDX3X plasmids.

C and D. The suppression of TNBC cell proliferation induced by ectopic expression of KLHL29 are rescued by re-expression of DDX3X in BT549 (C) and CAL51 (D) cells by cell viability assays using the Cell Counting Kit-8.

E-H. The suppression of TNBC cell migration and invasion induced by ectopic expression of KLHL29 are rescued by re-expression of DDX3X in BT549 and CAL51 cells by cell migration and invasion assays using transwell chambers coated without and with Matrigel, respectively. Representative images of migrated and invaded cells (E and G), and corresponding quantitative results (F and H) are shown.

***, $p < 0.001$ by two-tailed Student's t test or two-way ANOVA test.

Supplementary Fig. S6

A and B. Baf-A1 does not prevent KLHL29-mediated DDX3X degradation in BT549 (A) and CAL51 (B) cells. Cells expressing the empty vector pCDNA3.1-Flag or pCDNA3.1-Flag-KLHL29 plasmid were treated with DMSO or 200 nM Baf-A1 for 6 h before harvested for western blotting analysis.

C and D. Exogenous KLHL29 interacts with exogenous CUL3. BT549 cells were transfected with plasmids encoding Flag-KLHL29 and HA-CUL3, co-IP and western blotting assays.

E. CUL3 interacts with the BTB domain of KLHL29 in BT549 cells.

F. mRNA expression levels of CUL3 in 360 TNBC tissues and 88 adjacent normal tissues in FUSCC-TNBC RNA-seq dataset.

G. mRNA expression levels of CUL3 in 88 TNBC tissues and paired adjacent normal breast tissues in FUSCC-TNBC RNA-seq dataset.

H. mRNA expression levels of CUL3 in 169 TNBC tissues and 113 adjacent normal tissues in TCGA TNBC RNA-seq dataset.

I. mRNA expression levels of CUL3 in 13 TNBC tissues and paired adjacent normal breast tissues in TCGA TNBC RNA-seq dataset.

Data are represented as mean ± SD. Wilcoxon rank-sum test.

J and K. Ablation of CUL3 prevent KLHL29-mediated DDX3X degradation in BT549 (J) and CAL51 (K) cells. Cells expressing the empty vector pCDNA3.1-Flag or pCDNA3.1-Flag-KLHL29 were transfected with siNC and siRNAs targeting CUL3 before harvested for western blotting analysis.

L. Ectopic expression of KLHL29, but not KLHL29-ΔBTB, enhances the interaction of CUL3 and DDX3X. BT549 cells were transfected with plasmids encoding Myc-DDX3X and HA-CUL3 together with Flag-KLHL29-ΔBTB or Flag-KLHL29, followed by co-IP and western blotting assays.

Supplementary Fig. S7

A and B. Western blotting analysis showing the protein levels of Flag- KLHL29-ΔBTB and Flag-KLHL29 in BT549 (A) and CAL51 (B) cells.

C and D. Overexpression of KLHL29-ΔBTB does not influence cell proliferation of BT549 (C) and CAL51 (D) cells by cell viability assays using the Cell Counting Kit-8.

E-H. Overexpression of KLHL29-ΔBTB does not influence cell migration and invasion of BT549 and CAL51 cells by cell migration and invasion assays using transwell chambers coated without and with Matrigel, respectively. Representative images of migrated and invaded cells (E and G), and corresponding quantitative results (F and H) are shown.

***, $p < 0.001$, ns, no significance by two-tailed Student's t test or two-way ANOVA test.

Supplementary Fig. S8

A. Enrichment plot of the cell cycle pathway after DDX3X knockdown in SUM159PT cells by GSEA analysis.

B. KEGG pathway analysis of differentially expressed genes between cells silencing negative control and DDX3X in MDA-MB-231 and SUM159PT cells.

C and D. GO pathway analysis of differentially expressed genes between cells silencing negative control and DDX3X in MDA-MB-231 (C) and SUM159PT (D) cells.

E and F. Depletion of DDX3X represses the mRNA expression of CCNE1 and CCNA2 in MDA-MB-231 (E) and SUM159PT (F) cells by RT-qPCR analysis.

G and H. Ectopic expression of KLHL29 represses the mRNA expression of CCNE1 and CCNA2 in BT549 (G) and CAL51 (H) cells by RT-qPCR analysis.

I and J. Western blotting analysis showing the protein levels of DDX3X in MDA-MB-231 (I) and SUM159PT (J) cells treated with RK33.

*, $p < 0.05$, **, $p < 0.01$, ***, $p < 0.001$, ns, no significance by two-tailed Student's t test.

Supplementary Fig. S9

A and B. Dose–response curves and half maximal inhibition concentration values of RK33 in MDA-MB-231 (A) and SUM159PT (B) cells silencing negative control and DDX3X. Higher levels of DDX3X exhibit higher sensitivity to RK33.

C and D. Dose–response curves and half maximal inhibition concentration values of RK33 in BT549 (C) and CAL51 (D) cells expressing empty vector pCDNA3.1-Flag or pCDNA3.1-Flag-KLHL29 plasmids. Lower levels of KLHL29 exhibit higher sensitivity to RK33.

E and F. Higher levels of DDX3X indicate higher sensitivity of TNBC toward RK33. Representative images of organoids after RK33 treatment (E), organoid cell viability evaluated by CellTiter-Glo 3D Cell viability assay (F) are shown.

G and H. Lower levels of KLHL29 indicate higher sensitivity of TNBC toward RK33. Representative images of organoids after RK33 treatment (G), organoid cell viability evaluated by CellTiter-Glo 3D Cell viability assay (H) are shown.

***, $p < 0.001$ by two-tailed Student's t test.

Supplementary Fig. S10

A-D. Treatment with RK33 or carboplatin alone reduces cell growth of MDA-MB-231 (A), SUM159PT (B), BT549 (C), and CAL51 (D) cell lines compared with vehicle treatment by cell viability assays using the Cell Counting Kit-8. The combination of RK33 and carboplatin impairs TNBC cell growth compared with each of the individual treatments.

E and F. RK33 and carboplatin synergistically suppresses the growth of MDA-MB-231 cells. Cells were treated with combinations of agents, followed by the cell viability assays (E) and the Chou-Talalay analysis (F).

G and H. RK33 and carboplatin synergistically suppresses the growth of SUM159PT cells.

I and J. RK33 and carboplatin synergistically suppresses the growth of BT549 cells.

K and L. RK33 and carboplatin synergistically suppresses the growth of CAL51 cells.

M-P. The combination of RK33 and carboplatin dramatically suppresses the growth of MDA-MB-231 cell-derived orthotopic xenograft tumors of BALB/c female nude mice ($n = 7$ mice per group). Representative tumor images (M), tumor growth rate (N), tumor weight (O) and body weight (P) are shown.

Q and R. The combination of RK33 and carboplatin dramatically suppresses the growth of organoids derived from TNBC patients. Representative images of organoids after drug treatment (Q), and organoid cell viability evaluated by CellTiter-Glo 3D Cell viability assay (R) are shown.

*, $p < 0.05$, **, $p < 0.01$, ***, $p < 0.001$ by two-tailed Student's t test or two-way

ANOVA test.

Supplementary Fig. S11

A and B. The combination of RK33 and paclitaxel inhibits cell growth of MDA-MB-231 (A) and SUM159PT (B) than RK33 single-agent treatment, but shows comparable therapeutic efficacy with paclitaxel single-agent treatment.

C and D. The combination RK33 and paclitaxel shows no synergistic effects in MDA-MB-231 cells. Cells were treated with combinations of agents, followed by the cell viability assays (C) and the Chou-Talalay analysis (D).

E and F. The combination RK33 and paclitaxel shows no synergistic effects in SUM159PT cells.

*, $p < 0.05$, **, $p < 0.01$, ***, $p < 0.001$, no significance by two-tailed Student's t test or two-way ANOVA test.

Supplementary Fig. S1

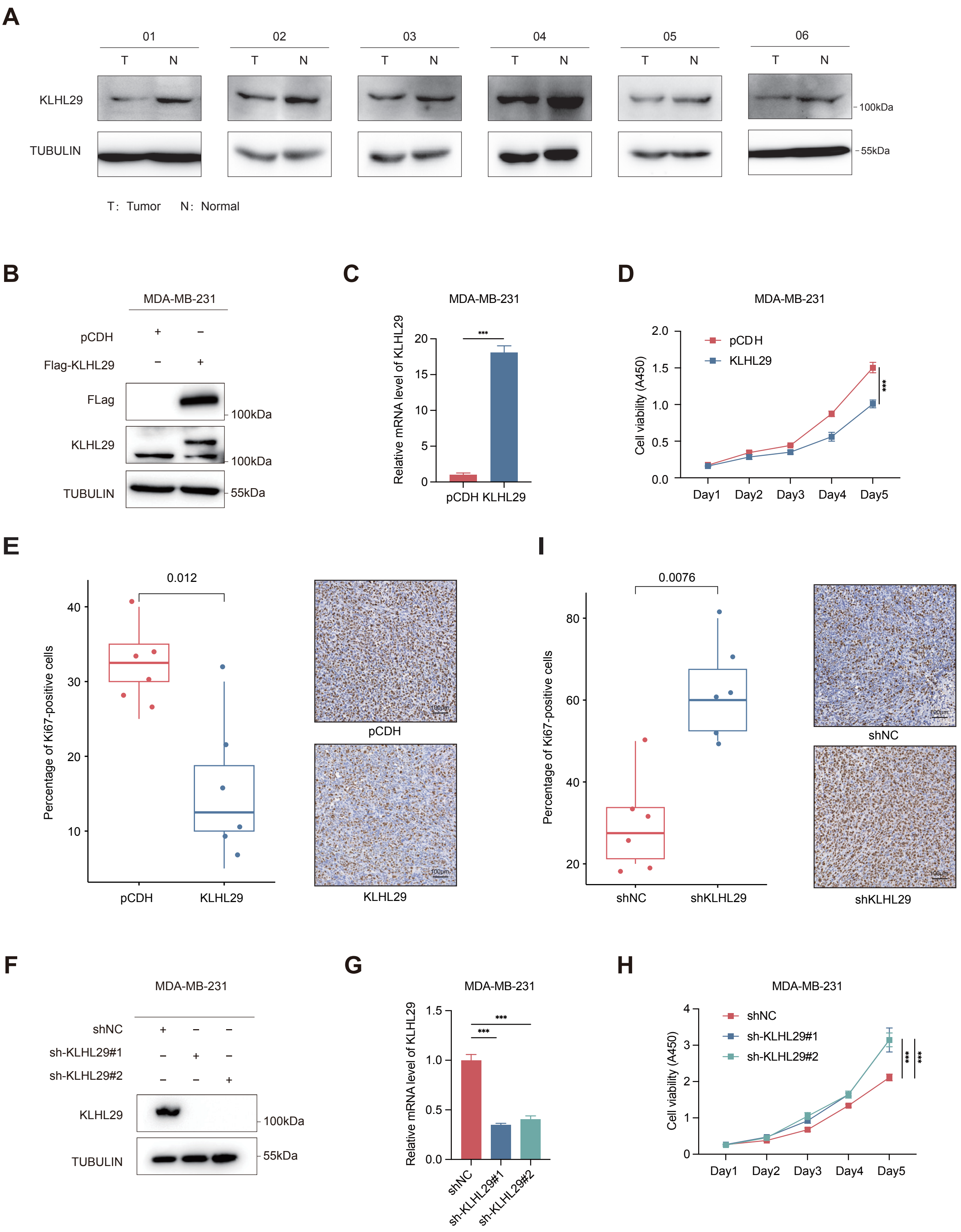

Supplementary Fig. S2

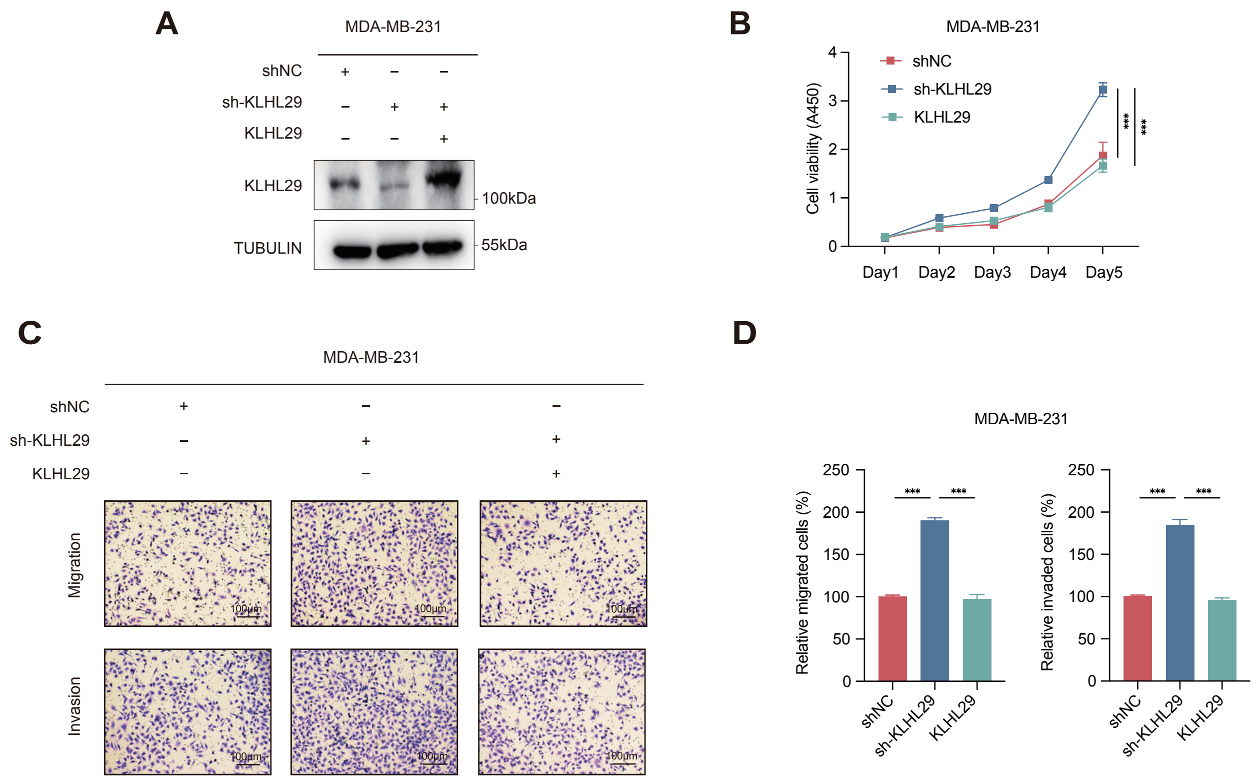

Supplementary Fig. S3

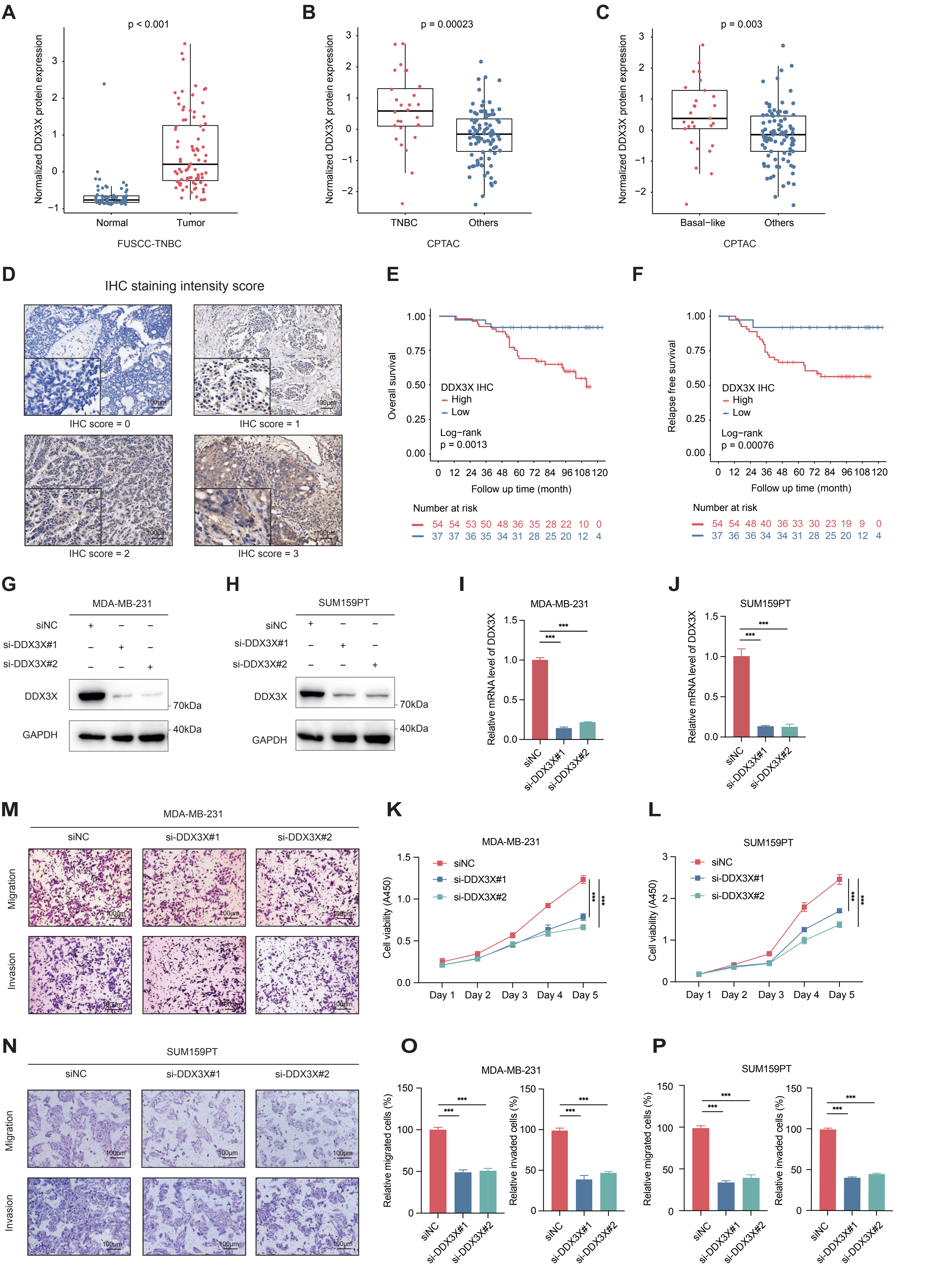

Supplementary Fig. S4

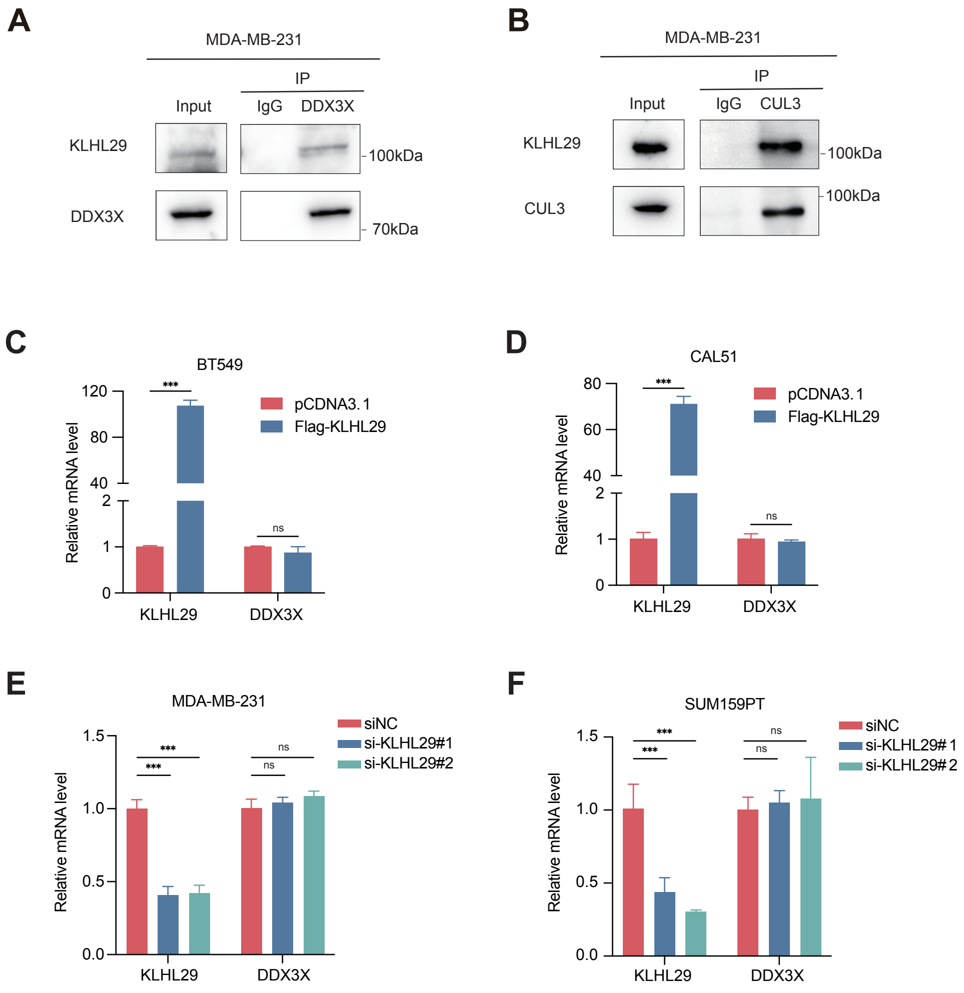

Supplementary Fig. S5

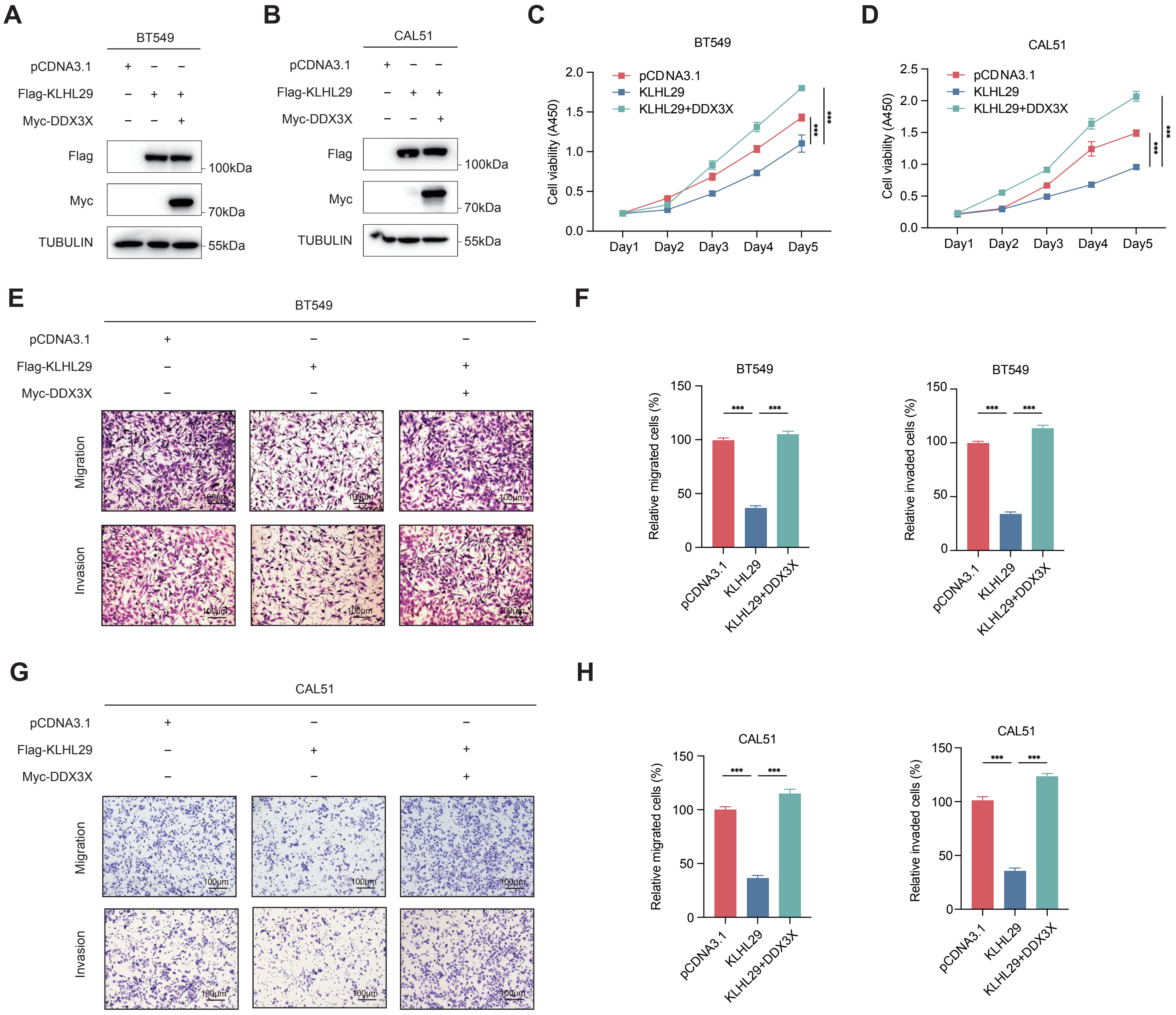

Supplementary Fig. S6

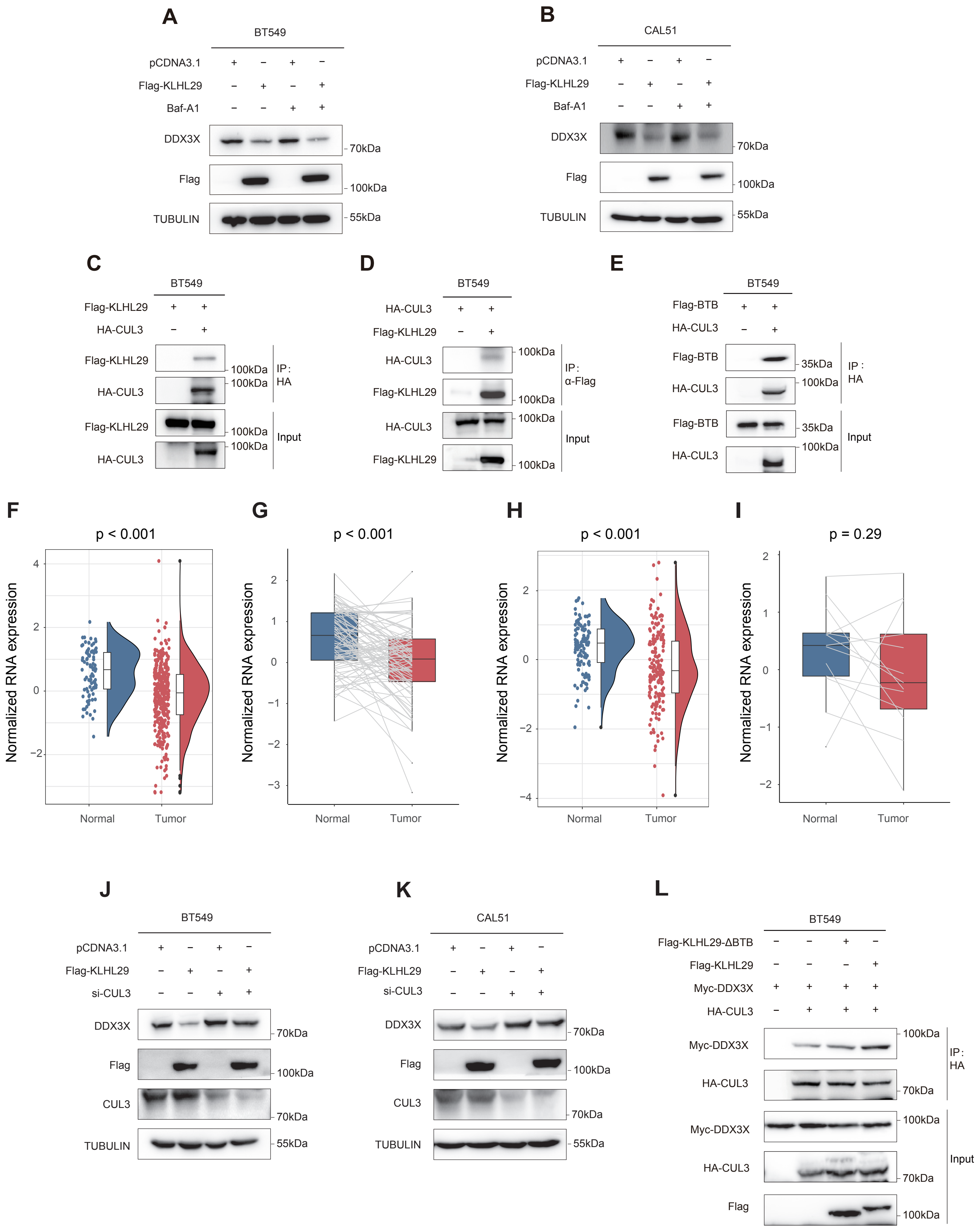

Supplementary Fig. S7

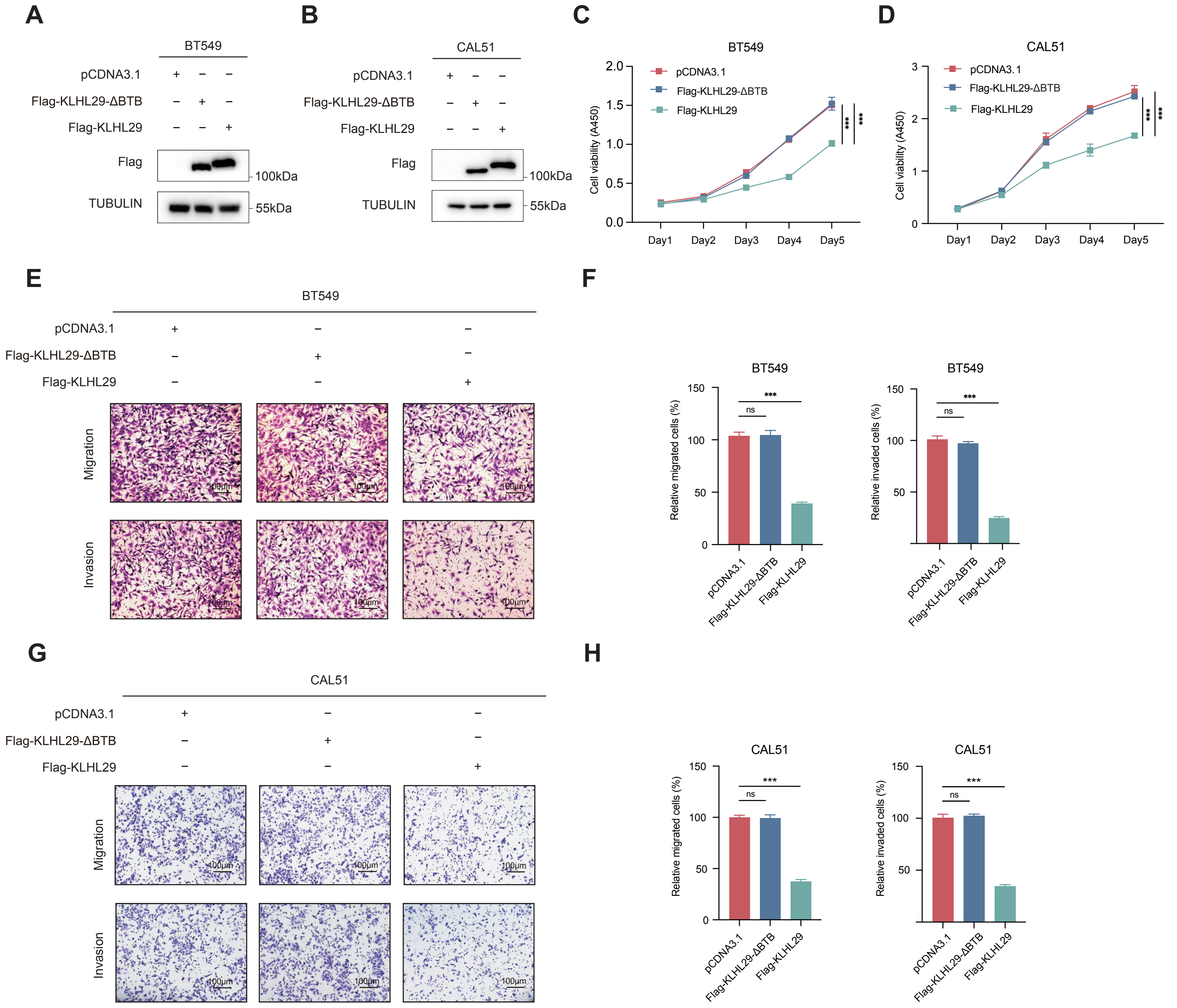

Supplementary Fig. S8

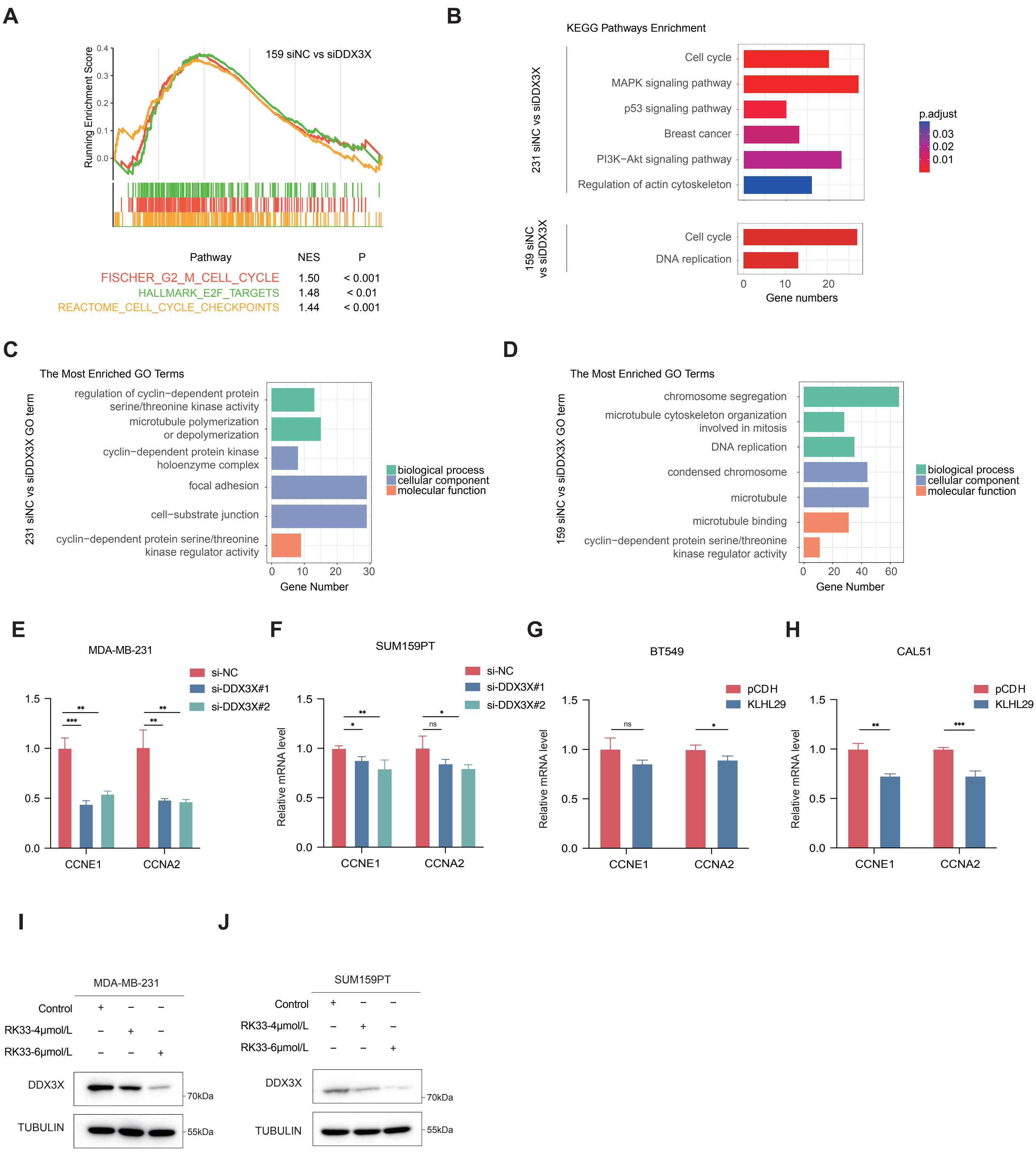

Supplementary Fig. S9

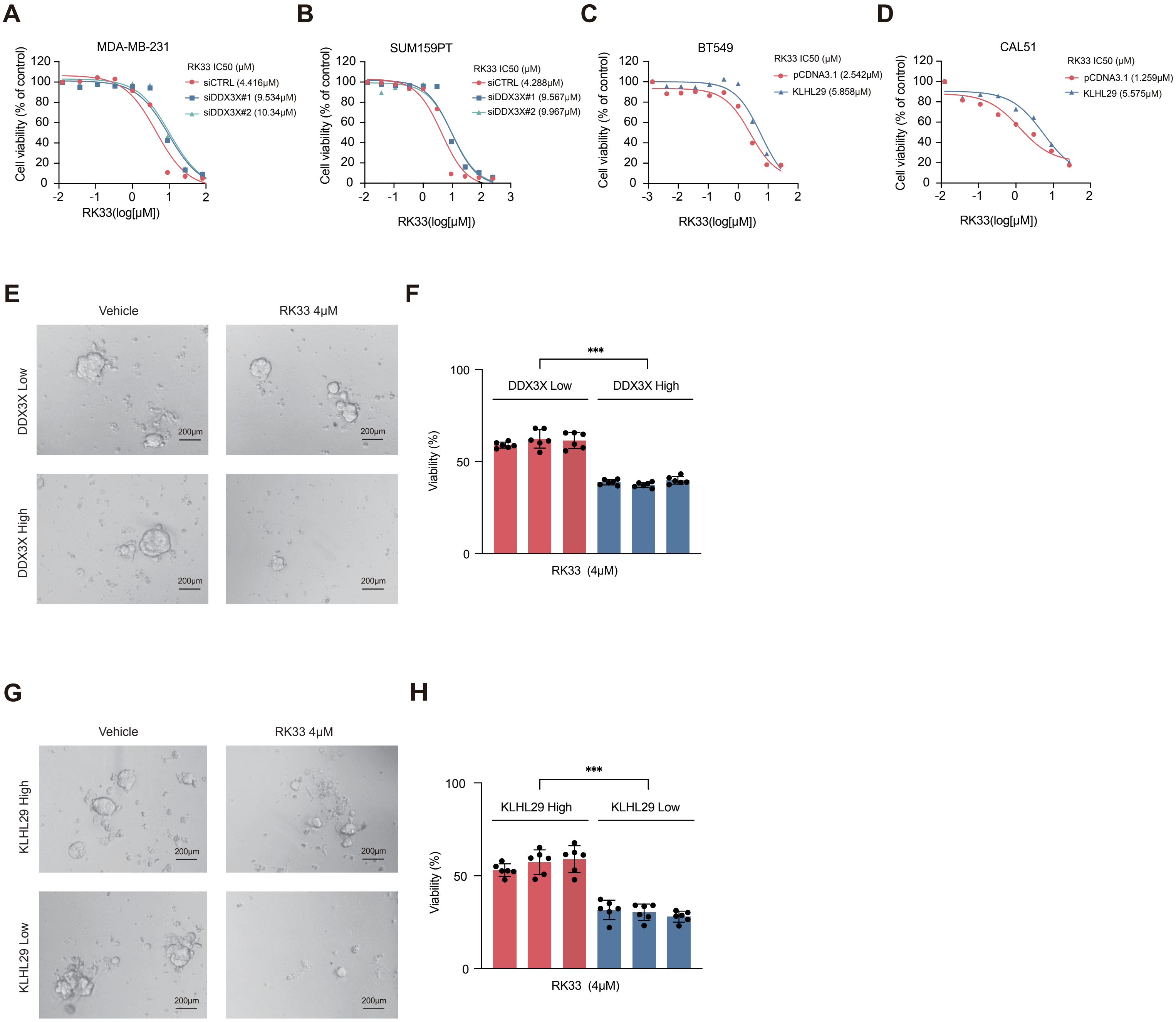

Supplementary Fig. S10

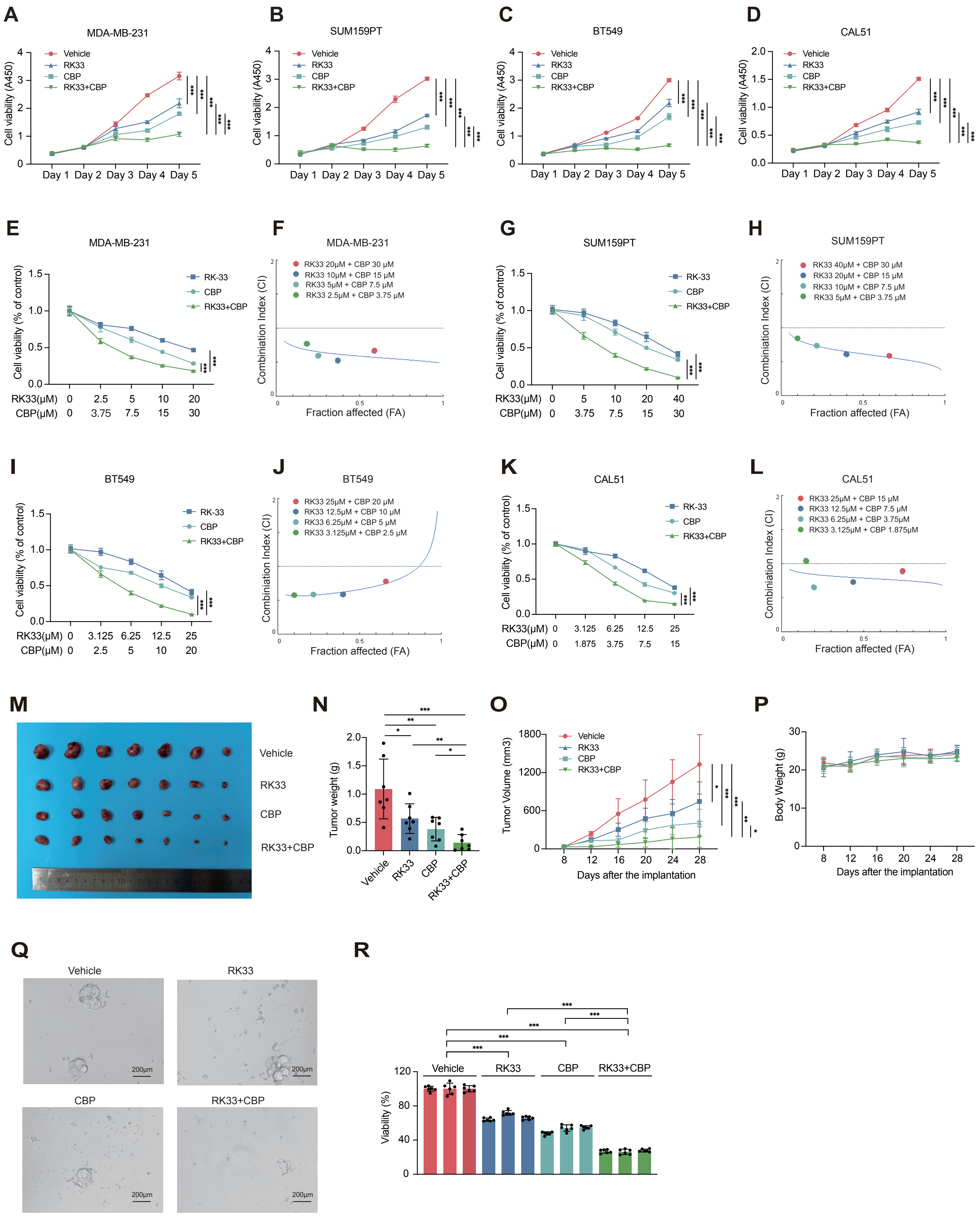

Supplementary Fig. S11

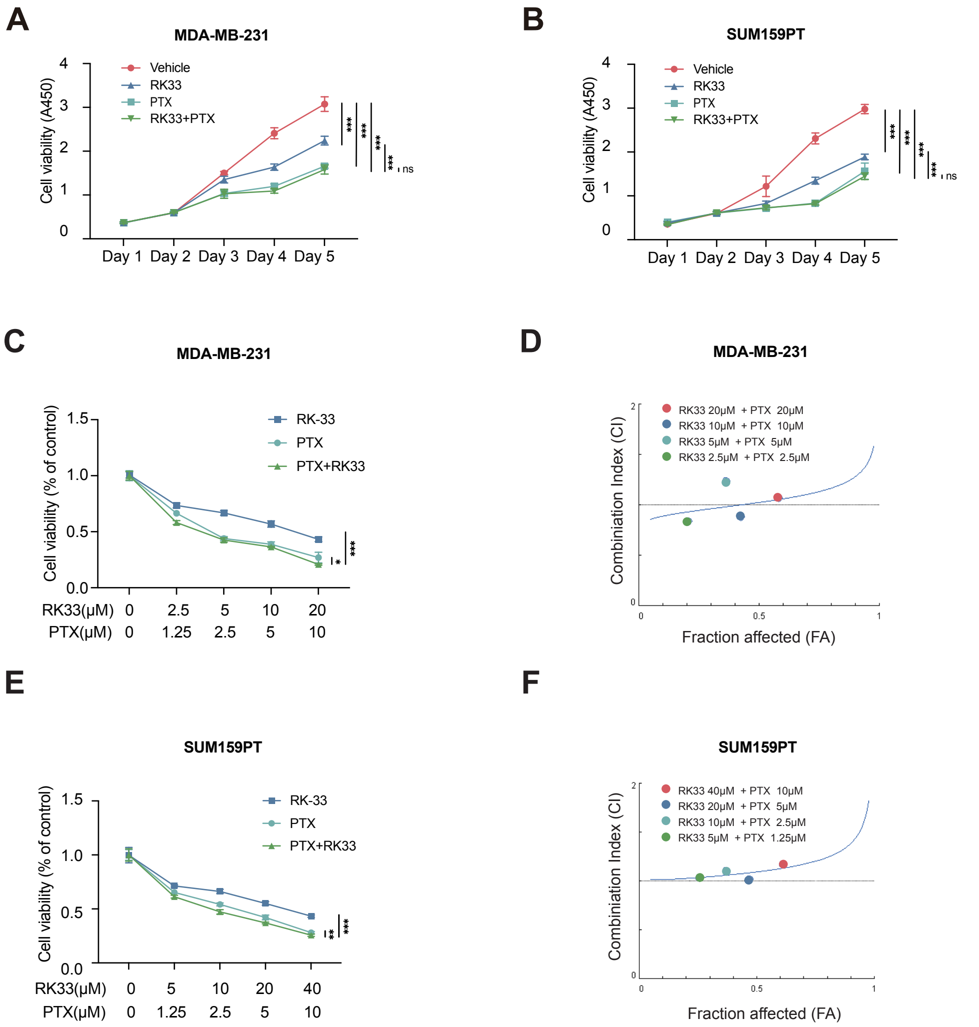

Supplementary Table S1 Univariate and multivariate Cox regression analyses of KLHL29 for overall survival in TNBC patients.

OS	Univariate Cox		Multivariate Cox	
	HR （95% CI）	P value	HR （95% CI）	P value
KLHL29 IHC score Low vs. High	5.67 （1.94-16.60）	0.002	4.99 （1.68-14.83）	0.004
Age ≥50y vs. <50y	2.99 （1.12-7.98）	0.029	3.23 （1.20-8.69）	0.020
Tumor size ≥2cm vs. <2cm	1.01 （0.42-2.43）	0.980	1.00 （0.41-2.42）	0.999
Lymph node N1-3 vs. N0	3.12 （1.33-7.32）	0.009	2.50 （1.05-5.95）	0.039

Supplementary Table S2 Univariate and multivariate Cox regression analyses of KLHL29 for relapse-free survival in TNBC patients.

RFS	Univariate Cox		Multivariate Cox	
	HR （95% CI）	P value	HR （95% CI）	P value
KLHL29 IHC score<br>Low vs. High	5.98<br>（2.06-17.38）	0.001	5.92<br>（2.02-17.32）	0.001
Age<br>≥50y vs. <50y	2.35<br>（0.94-5.86）	0.067	2.70<br>（1.08-6.80）	0.034
Tumor size<br>≥2cm vs. <2cm	0.95<br>（0.41-2.18）	0.898	0.93<br>（0.40-2.17）	0.872
Lymph node<br>N1-3 vs. N0	2.43<br>（1.08-5.47）	0.032	2.18<br>（0.96-4.97）	0.063

Supplementary Table S3 Univariate and multivariate Cox regression analyses of DDX3X for overall survival in TNBC patients.

OS	Univariate Cox		Multivariate Cox	
	HR（95% CI）	P value	HR（95% CI）	P value
DDX3X IHC score High vs. Low	5.71（1.71-19.10）	0.005	6.78（1.98-23.16）	0.002
Age ≥50y vs. <50y	2.99（1.12-7.98）	0.029	4.04（1.48-11.01）	0.006
Tumor size ≥2cm vs. <2cm	1.01（0.42-2.43）	0.98	1.27（0.52-3.10）	0.597
Lymph node N1-3 vs. N0	3.12（1.33-7.32）	0.009	3.69（1.54-8.84）	0.003

Supplementary Table S4 Univariate and multivariate Cox regression analyses of DDX3X for relapse-free survival in TNBC patients.

RFS	Univariate Cox		Multivariate Cox	
	HR（95% CI）	P value	HR（95% CI）	P value
DDX3X IHC score<br>High vs. Low	6.09<br>（1.83-20.31）	0.003	6.73<br>（2.00-22.71）	0.002
Age<br>≥50y vs. <50y	2.35<br>（0.94-5.86）	0.067	2.85<br>（1.13-7.20）	0.027
Tumor size<br>≥2cm vs. <2cm	0.95<br>（0.41-2.18）	0.898	1.09<br>（0.47-2.53）	0.849
Lymph node<br>N1-3 vs. N0	2.43<br>（1.08-5.47）	0.032	2.86<br>（1.25-6.57）	0.013

Supplementary Table S5 Primers for construction and others.

Primer	Sequences (5'-3')
pCDNA3.1-Flag-KLHL29-F-EcoRI	CGGAATTCATATGTCCCGGCACCATAGC
pCDNA3.1-Flag-KLHL29-R-XbaI	GCTCTAGATTAGTGGTGGTGGTGGTGGT
pCDH-KLHL29-F-XbaI	GCTCTAGAATGTCCCGGCACCATAGC
pCDH-KLHL29-R-EcoRI	CGGAATTCTTAGTGGTGGTGGTGGTGGTGCT
pCDNA3.1-KLHL29-(1 to 328)-F-EcoRI	CGGAATTCATATGTCCCGGCACCATAGC
pCDNA3.1-KLHL29-(1 to 328)-R-XbaI	GCTCTAGATTATTAAAACGCTTTCGCTCTGCGTTGCTG
pCDNA3.1-KLHL29-(1 to 401)-F-EcoRI	CGGAATTCATATGTCCCGGCACCATAGC
pCDNA3.1-KLHL29-(1 to 401)-F-XbaI	GCTCTAGATTATTACGAGTCGATGACCAGGGAGC
pCDNA3.1-KLHL29-(329 to 584)-F-EcoRI	CGGAATTCATACAGACCTGAAAATTGTTG
pCDNA3.1-KLHL29-(329 to 584)-F-XbaI	GCTCTAGATTATTACTCAGCCACACCTGCAGA
pCDNA3.1-KLHL29-(585 to 875)-F-EcoRI	CGGAATTCATGTCATCGTCTTGGTTGGGGG
pCDNA3.1-KLHL29-(585 to 875)-F-XbaI	GCTCTAGATTATTAGCCGCTTTGAATATATTT
pCDNA3.1-KLHL29-del-BTB-F	GCCAACGCCAAGACACTGCTGG
pCDNA3.1-KLHL29-del-BTB-R	AAACGCTTTCGCTCTGCGTTG
sh-KLHL29-F1	CCGGTCGTCTACGATGGGAAGATTTCTCGAGAAATCTTCCCATCGTAGACGATTTTTG

sh-KLHL29-R1	AATTCAAAAATCGTCTACGATGGGAAGATTTCTCGAGAAATCTTCCCATCGTAGACGA
sh-KLHL29-F2	CCGGAGAGCAAGCTTGATCCCTAAACTCGAGTTTAGGGATCAAGCTTGCTCTTTTTG
sh-KLHL29-R2	AATTCAAAAAGAGCAAGCTTGATCCCTAAACTCGAGTTTAGGGATCAAGCTTGCTCT
pCDNA3.1-Myc/His-CUL3-F-NheI	CTAGCTAGCATGTCGAATCTGAGCAAA
pCDNA3.1-Myc/His-CUL3-F-XhoI	CCGCTCGAGTTATTAGGCATAATCGGGTACATCGTAAGGGTA

Supplementary Table S6 Sequences of siRNAs.

siRNA	Sequences (5’-3’)
siNC	UUCUCCGAACGUGUCACGU
siKLHL29-1	CUACGACCCUGAGAAAGGA
siKLHL29-2	GAGAAAUGUUGAAGGAAUUGA
siDDX3X-1	CGAGAGAGUUGGCAGUACA
siDDX3X-2	CAUUGAGCUUACUCGUUAU
siCUL3	GAAGGAAUGUUUAGGGAUA

Supplementary Table S7 Primers for RT-qPCR.

Primer	Sequences（5’-3’）
GAPDH-F	GGAGCGAGATCCCTCCAAAAT
GAPDH-R	GGCTGTTGTCATACTTCTCATGG
ACTB-F	CATGTACGTTGCTATCCAGGC
ACTB-R	CTCCTTAATGTCACGCACGAT
KLHL29-1-F	GCAGAGCGAAAGCGTTTACAG
KLHL29-1-R	GCAGGTTCGACAGGACGAG
KLHL29-2-F	GGAACCCGCAGAACAACAAG
KLHL29-2-R	CCATCCCACCTGAGAGGTAGAT
DDX3X-1-F	ACGAGAGAGTTGGCAGTACAG
DDX3X-1-R	ATAAACCACGCAAGGACGAAC
CCND1-F	GCTGCGAAGTGGAAACCATC
CCND1-R	CCTCCTTCTGCACACATTTGAA
CCNA2-F	CGCTGGCGGTACTGAAGTC
CCNA2-R	GAGGAACGGTGACATGCTCAT
CCNE1-F	AAGGAGCGGGACACCATGA
CCNE1-R	ACGGTCACGTTTGCCTTCC

Supplementary Table S8 Antibodies used in this study.

Antibodies	Vendors	catalog number
Mouse IgG	Santa Cruz Biotechnology	sc-2025
Rabbit IgG	Cell Signaling Technology	2729S
Flag tag	Sigma-Aldrich	F1804
Myc tag (Rabbit)	Proteintech	16286-1-AP
Myc tag (Mouse)	Proteintech	60003-2-Ig
HA tag	Proteintech	51064-2-AP
GAPDH	Proteintech	10494-1-AP
α-tubulin	Proteintech	66031-1-Ig
KLHL29	Novus	NBP2-56832
KLHL29	Proteintech	20162-1-AP
DDX3X	Proteintech	11115-1-AP
CUL3	Proteintech	11107-1-AP
Ubiquitin	Santa Cruz Biotechnology	sc-8017
